# COVID-19 impact on mental health

**DOI:** 10.1186/s12874-021-01411-w

**Published:** 2022-01-14

**Authors:** Jingyu Cui, Jingwei Lu, Yijia Weng, Grace Y. Yi, Wenqing He

**Affiliations:** 1 Department of Statistical and Actuarial Sciences, Western University, London, Ontario N6A 5B7 Canada; 2 Department of Computer Science, Western University, London, Ontario N6A 5B7 Canada

**Keywords:** COVID-19, Lasso, logistic regression, mental health, missing data, multiple imputation, survey data

## Abstract

**Background:**

The coronavirus disease 2019 (COVID-19) pandemic has posed a significant influence on public mental health. Current efforts focus on alleviating the impacts of the disease on public health and the economy, with the psychological effects due to COVID-19 relatively ignored. In this research, we are interested in exploring the *quantitative* characterization of the pandemic impact on public mental health by studying an online survey dataset of the United States.

**Methods:**

The analyses are conducted based on a large scale of online mental health-related survey study in the United States, conducted over 12 consecutive weeks from April 23, 2020 to July 21, 2020. We are interested in examining the risk factors that have a significant impact on mental health as well as in their estimated effects over time. We employ the multiple imputation by chained equations (MICE) method to deal with missing values and take logistic regression with the least absolute shrinkage and selection operator (Lasso) method to identify risk factors for mental health.

**Results:**

Our analysis shows that risk predictors for an individual to experience mental health issues include the pandemic situation of the State where the individual resides, age, gender, race, marital status, health conditions, the number of household members, employment status, the level of confidence of the future food affordability, availability of health insurance, mortgage status, and the information of kids enrolling in school. The effects of most of the predictors seem to change over time though the degree varies for different risk factors. The effects of risk factors, such as States and gender show noticeable change over time, whereas the factor age exhibits seemingly unchanged effects over time.

**Conclusions:**

The analysis results unveil evidence-based findings to identify the groups who are psychologically vulnerable to the COVID-19 pandemic. This study provides helpful evidence for assisting healthcare providers and policymakers to take steps for mitigating the pandemic effects on public mental health, especially in boosting public health care, improving public confidence in future food conditions, and creating more job opportunities.

**Trial registration:**

This article does not report the results of a health care intervention on human participants.

**Supplementary Information:**

The online version contains supplementary material available at 10.1186/s12874-021-01411-w.

## Background

Since the outbreak of the COVID-19 pandemic, people's lifestyle has been changed significantly. However, no sufficient resources have been available to attenuate the pandemic effects on mental health and well-being [1]. Various studies have been conducted to investigate how the COVID-19 pandemic may affect people psychologically. For example, Cao et al. [2] conducted a survey on college students in China and showed that more than 24% of the students were experiencing anxiety. Spoorthy et al. [3] investigated the mental health problems faced by healthcare workers during the COVID-19 pandemic.

While those studies provided descriptive results by summarizing the information obtained from the questionnaire, it is unclear how the impact of COVID-19 changes over time; what factors are relevant to describe the impact of the pandemic; and how the severity of the mental health issues is quantitatively associated with the risk factors. In this paper, we examine these questions and aim to provide some quantitative insights. Our explorations are carried out using a large scale online public survey study conducted by the *U.S. Census Bureau* [4]. The data include twelve data sets each collected in a 1-week window over 12 consecutive weeks from April 23, 2020 to July 21, 2020. Different data sets contain the measurements from different participants on the same questions. Among the 12 data sets, the smallest one contains 41,996 subjects and the largest one has 132,961 participants. We treat the survey in each week as an independent study. We are interested in assessing how the effects of the associated risk factors may change over time by applying the same method to each of the 12 data sets separately.

The survey includes multiple questions perceived to be relevant to describing the impact of the pandemic on the public. To quantitatively identify the risk factors for impacting the mental health by the pandemic, we engage the penalized logistic regression method, with the least absolute shrinkage and selection operator (Lasso) penalty [5]. However, a direct application of the Lasso method is not possible due to the presence of missing observations. To handle missing values, we employ the *multiple imputation by chained equations* (MICE) method (e.g., [6, 7]). Further, survey data commonly involve measurement error due to recall bias, the inability of providing precise descriptions of some answers, and reporting errors. It is imperative to address this issue when pre-processing the data. To this end, we combine the levels of those highly related categorical variables to mitigate the measurement error effects.

## Methods

### Original survey data

The data used in this project are from phase 1 of the Household Pulse Survey conducted by the *U.S. Census Bureau* [4] from April 23, 2020 to July 21, 2020 for 12 consecutive weeks, giving rise to 12 data sets each for a week. The survey aims to study the pandemic impacts on the households across the United States from social and economic perspectives. The survey contains 50 questions ranging from education, employment, food sufficiency, health, housing, social security benefits, household spending, stimulus payments, to transportation. The participants of the survey come from all the 50 states plus Washington, D.C., United States, aging from 18 to 88. The gender ratio (the ratio of males to females) remains fairly stable ranging between 0.6 and 0.7 over the 12 weeks. Figure S1 in the Supplementary Material shows the curves of the number of cumulative confirmed cases for all the states which are grouped into four categories of the severity of the pandemic, derived from the data from the *Centers for Disease Control and Prevention* [8]. Table 1 lists the state members for each category, together with the total number of participants over the 12 weeks and the corresponding percentage for each category. It is seen that the majority (72.5%) of the participants of the survey come from the states with mild pandemic and the least proportion (2.3%) of subjects are from the states with a serious pandemic.

**Table 1:** Classification of the States According to the Severity of Pandemic

Severity of Pandemic	States	Total Number of Participants over 12 weeks (Percentage)
Mild Pandemic	Alabama, Alaska, Arkansas, Colorado, Connecticut, Delaware, District of Columbia, Hawaii, Idaho, Indiana, Iowa, Kansas, Kentucky, Louisiana, Maine, Maryland, Massachusetts, Michigan, Minnesota, Mississippi, Missouri, Montana, Nebraska, Nevada, New Hampshire, New Mexico, North Carolina, North Dakota, Ohio, Oklahoma, Oregon, Pennsylvania, Rhode Island, South Carolina, South Dakota, Tennessee, Utah, Vermont, Virginia, Washington, West Virginia, Wisconsin, Wyoming	748165 (72.5%)
Moderate Daily Increase	Arizona, Georgia, Illinois, New Jersey	94886 (9.2%)
Large Daily Increase	California, Florida, Texas	165982 (16.0%)
Serious Pandemic	New York	23310 (2.3%)

The States are classified into four categories (*mild*, *moderate, large daily increase,* and *serious*) according to the severity of the pandemic. The last column records the total number of participants in the survey over 12 weeks and the corresponding percentage for each category

### Pre-processing the data to reduce errors

Among the initial 50 questions, nine questions, such as “*Where did you get free groceries or free meals*” and “*How often is the Internet available to children for educational purposes*”, are excluded because they are not perceived as sustainable factors on affecting mental health. Measurement error is typically involved in survey data. Prior to a formal analysis of the data, we implement a pre-processing procedure to mitigate the measurement error effects by combining questions to create new variables, or collapsing levels of variables to form binary variables.

Information on mental health is collected via four questions concerning *anxiety*, *worry*, *loss of interest*, and *feeling down*. Each question is a four-level Likert item [9] with values 1, 2, 3 and 4, showing the degree of each aspect for the past 7 days prior to the survey time. In contrast to Twenge and Joiner [10] who combined the measurements of the first two questions *anxiety* and *worry* to indicate the anxiety level and the last two questions *loss of interest* and *feeling down* to show the depression level, we define a single binary response to reflect the mental health status of an individual by combing measurements of the four variables. The response variable takes value 1 if the average of the scores of the four variables is greater than 2.5, and 0 otherwise, where the threshold 2.5 is the median value for each question. This binary response gives a synthetic way to indicate the mental health status which is easier thaeach question. This binary response gives a synthetic wayn examining measurements of multiple variables.

Two variables describe the loss of work: *Wrkloss* indicates whether an individual in the household experiences a loss of employment income since March 13, 2020; *Expctloss* indicates if the individual expects a member in the household to experience a loss of employment income in the next 4 weeks because of the COVID-19 pandemic. These two variables are combined to form a single indicator which is denoted *Wrkloss*, with value 1 if at least one of these two events happens. Two ordinal variables, *Prifoodsuf* and *Curfoodsuf*, are used to describe the food sufficiency status before the pandemic and at present, respectively. The *Foodcon.change* variable is constructed by comparing the current and the previous food sufficiency status to form a binary variable, taking 1 if the current food sufficiency status is no worse than the food status before the pandemic, and 0 otherwise. Variable *Med.delay.notget* is combined from two indicator variables *Delay* (indicating if medical care is delayed) and *Notget* (indicating if the medical care is not received), taking value 1 if either medical care is delayed or no medical care is received, and 0 otherwise. Predictor *Mort.prob* is combined from one binary variable and an ordinal variable, taking 1 if a participant does not pay last month’s rent or mortgage or does not have enough confidence in paying the next rent or mortgage payment on time, and 0 otherwise. In addition, three ordinal variables, *Emppay*, *Healins,* and *Schoolenroll*, are modified by collapsing their levels to form binary categories. *Emppay* has value 1 if he/she gets paid for the time he/she is not working, and 0 otherwise. *Healins* has value 1 if the individual is currently covered by the health insurance, and 0 otherwise. *Schoolenroll* has value 1 if there is a child in the household enrolled in school, and 0 otherwise. Except for the variables discussed above, the remaining variables are kept as in the original form.

The final data include the binary response (indicating the mental health status of an individual) and 25 predictors measuring various aspects of individuals. To be specific, nine predictors show basic information: *State*, *Age*, *Male*, *Rhispanic*, *Race*, *Educ*, *MS* (marital status), *Numper* (the number of people in the household), and *Numkid* (the number of people under 18 in the household); five variables concern the income and employment: *Income*, *Wrkloss*, *Anywork*, *Kindwork*, and *Emppay*; five variables are related to food: *Foodcon.change*, *Freefood*, *Tspndfood*, *Tspndprpd*, and *Foodconf*; three variables pertain to health and insurance: *Hlthstatus*, *Healins*, and *Med.delay.notget*; one variable, *Mort.prob*, is for mortgage and housing; and two variables, *Schoolenroll* and *Ttch_Hrs*, reflect child education. The variable dictionary for the pre-processed data is shown in Table 2.

**Table 2:** Variable Description

Variable	Description
State	A categorical variable indicating the pandemic severity of the state from which a participant comes. Four levels are classified as *mild*, *moderate daily increase* (the states that have moderate daily new cases), *large daily increase* (the states that have large daily new cases), and *serious*.
Age	Age of a participant, ranging from 18 to 88.
Male	The gender indicator. 1: male; 0: female.
Rhispanic	The indicator of Hispanic, Latino or Spanish origin. 1: yes; 2: no.
Race	A categorical variable with four levels. 1: White; 2: Black; 3: Asian; 4: others.
Educ	The highest degree of education completed. 1: less than high school; 2: high school; 3: Bachelor’s degree; 4: graduate degree.
MS	A categorical variable with 5 levels indicating marital status. 1: now married; 2: widowed; 3: divorced; 4: separated; 5: never married.
Numper	The total number of household members including adults and children.
Numkid	The total number of household members under 18 years.
Income	A categorical variable of the total household income in 2019 before taxes. 1: Less than $25,000; 2: $25,000 - $34,999; 3: $35,000 - $49,999; 4: $50,000 - $74,999; 5: $75,000 - $99,999; 6: $100,000 - $149,999; 7: $150,000 - $199,999; 8: $200,000 and above.
Wrkloss	An indicator variable indicating if anyone in the household experienced a loss of employment income since March 13, 2020, or the participants expect anyone in the household will experience a loss of employment income in the next 4 weeks because of the coronavirus pandemic. 1: yes; 2: no.
Anywork	An indicator variable indicating if the participant did any work for either pay or profit in the last 7 days. 1: yes; 2: no.
Kindwork	A categorical variable indicating the kind of work for the participant. 1: government; 2: private company; 3: non-profit organization including tax and charitable organizations; 4: self-employed; 5: working in a family business.
Emppay	An indicator variable indicating if the participant receives payment for the time not working. 1: yes; 2: no.
Foodcon.change	An indicator variable indicating how the food eaten status changes from before March 13, 2020 till now. 1: the food status is better or keeps the same; 0: the food status becomes worse.
Freefood	An indicator variable indicating if the participant or anyone in the household gets free groceries or a free meal during the last 7 days.
Tspndfood	A continuous variable showing the amount of money the household spends on food at supermarkets, grocery stores, online, etc., to buy food to prepare and eat at home.
Tspndprpd	A continuous variable showing the amount of money the household spends on prepared meals, including eating out, fast food, etc.
Foodconf	An ordinal variable with four levels indicating the confidence level that the household is able to afford the kind of food needed for the next four weeks. 1: not at all confident; 2: somewhat confident; 3: moderately confident; 4: very confident.
Hlthstatus	An ordinal variable indicating the health status: 1: excellent; 2: very good; 3: good; 4: fair; 5: poor.
Healins	An indicator variable indicating if the participant is currently covered by any type of health insurance or health coverage plan. 1: yes; 2: no.
Med.delay.notget	An indicator variable indicating if the medical care is delayed or not having medical care. 1: yes; 2: no.
Mort.prob	An indicator variable indicating if there is any problem on rental or mortgage, including last month’s rent or mortgage is not paid on time, or the participant does not have enough confidence in the ability to pay the next month rent or mortgage on time. 1: yes; 2: no.
Schoolenroll	An indicator variable indicating if there is any child in the household enrolled in the school. 1: yes; 2: no.
Ttch_Hrs	A continuous variable recording the hours that the household members spend on all teaching activities with children during the last 7 days.
Mental health	A binary variable with 1 indicating having mental health problem and 0 otherwise.

### Missing observations

**Fig. 1:**
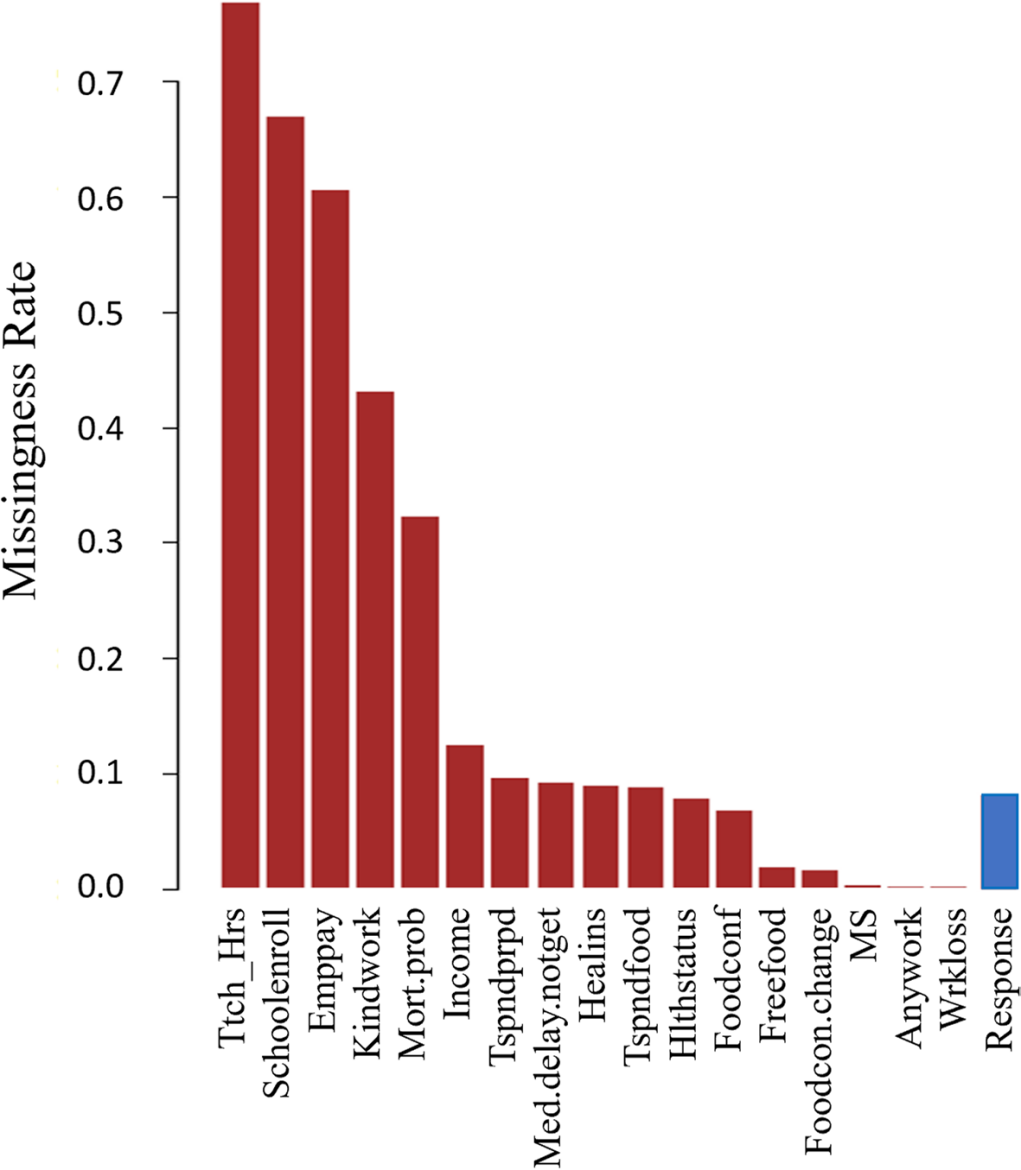
The *missingness rates* for the 17 risk factors and the response of the pooled data

In the data sets, 17 covariates together with the response variable have missing observations. To provide a quick and intuitive sense of the missingness proportions for different variables over the 12 data sets, we combine those data sets by individual variable to form a single pooled data set. Then we calculate the missingness proportion for each variable by dividing the number of missing observations in the variable by the total number of subjects in the pooled data set. We display in Fig. 1 the *missingness rates* for those 17 risk factors and the response variable (mental health status) for the pooled data. The risk factors having the three highest missingness rates are the variables *Ttch_hrs*, *Schoolenroll* and *Emppay*, and the corresponding missingness rates are 76.7%, 66.9% and 60.5%, respectively. Five variables incur higher than 30% missingness proportions, and the missingness proportion for 12 risk factors is larger than 5%. The missingness proportion for the response variable is about 8.6%.

Missing values present a challenge for data analysis and model fitting. One may perform the so-called *complete data analysis* by deleting those subjects with missing observations or the so-called *available data analysis* by using all available data, and then repeating a standard analysis procedure. Such analyses are easy to implement, however, biased results are expected if the *missing completely at random* (MCAR) mechanism is not true. Here we consider a broader setting where missing data do not necessarily follow the MCAR but follow the *missing at random* (MAR) mechanism. We employ the MICE method which is developed under the MAR mechanism and applies to various types of variables such as continuous, binary, nominal, and ordinal variables subject to missingness. A detailed discussion on this method was provided by van Buuren et al. [11].

Here we employ the MICE method to accommodate missing observations that are present in both the predictors and the response. Following the suggestion of Allison [12], we choose to do five imputations for the data in each week by employing the same algorithm with different random seeds. The implementation is conducted in R (version 3.6.1) with the R package: *Multivariate Imputation by Chained Equation* (mice). The details on the R code are presented in the *code availability* in the Declarations section**.**

To empirically assess the imputation results, we take the data in week 6 as an example and compare the five imputed data sets to the original data by displaying their distribution using the R function *density* for the continuous variables; the results are reported in Figure S2 in the Supplementary Material. It is seen that the distributions of the 5 imputed data sets for the three continuous variables, *Tspndfood*, *Tspndprpd*, and *Ttch_hrs*, are fairly similar to that of the original data. Further, in Tables S1, S2, and S3 in the Supplementary Material, we report the proportions of different levels for the categorical variables for both the imputed and original data, showing the similarity in the distributions of the imputed data and of the original data.

### Model building and inference

We intend to employ logistic regression with the Lasso penalty to analyze the data that contain a binary response and potentially related predictors or covariates. First, we introduce the basic notation and discuss the method in general terms. For *i* = 1, …, *n*, let *Y*_*i*_ represent the binary response with value 1 indicating that the mental health problem occurs for subject *i* and 0 otherwise. Let *X*_*ij*_ denote the *j*th covariate for subject *i*, where *j* = 1, …, *p*, and *p* is the number of predictors. Write *X*_*i*_ = (*X*_*i*1_, *X*_*i*2_, …, *X*_*ip*_)^T^ and let *π*_*i*_ = *P*(*Y*_*i*_ = 1| *X*_*i*_).

Consider the logistic regression model

1 $$\mathrm{logit}\;\pi_i=\beta_0+X_i^T\beta,$$

where *β* = (*β*_1_, …,*β*_*p*_)^T^ denotes the vector of regression parameters. Consequently, the log-likelihood function for *β* is given by

2 $${\displaystyle \begin{array}{c}l\left(\beta \right)=\sum_{i=1}^n\left[{Y}_i\log {\pi}_i+\left(1-{Y}_i\right)\log \left(1-{\pi}_i\right)\right]\\ {}=\sum_{i=1}^n\left[{Y}_i\left({\beta}_0+{X}_i^{\mathrm{T}}\beta \right)-\log \left\{1+\exp \left({\beta}_0+{X}_i^{\mathrm{T}}\beta \right)\right\}\right].\end{array}}$$

To select the predictors associated with the dichotomous response, we employ the Lasso method. The Lasso estimates are the values that maximize the penalized log-likelihood function obtained by adding an *L*_1_ penalty to the expression (2):

3 $${l}_{\lambda}^{Lasso}\left(\beta \right)=\sum_{i=1}^n\left[{Y}_i\left({\beta}_0+{X}_i^{\mathrm{T}}\beta \right)-\log \left\{1+\exp \left({\beta}_0+{X}_i^{\mathrm{T}}\beta \right)\right\}\right]+\lambda \sum_{j=1}^p\mid {\beta}_j\mid,$$

where *λ* is the tuning parameter. The 10-fold cross-validation is employed to obtain a proper value for the tuning parameter and the *one-standard-error* rule [13] is applied to pick the most parsimonious model within one standard error of the minimum cross-validation misclassification rate (e.g., [14]).

### Model fitting and variable selection

The Lasso logistic regression is applied to each of the five imputed data sets for each week. The predictors corresponding to the nonzero coefficient estimates are considered the risk factors selected, which may be different across five imputed data sets for each of the 12 weeks. To explore in a full spectrum, we start with two extreme models, called the *full model* by including the union of all the selected risk factors by the Lasso logistic regression, and the *reduced model* by including only the common factors selected for all five imputed data sets in any week. The *full model* includes all the 25 predictors in the original data, and the *reduced model* contains 11 predictors: *Age*, *Male*, *MS*, *Numkid*, *Wrkloss*, *Anywork*, *Foodconf*, *Hlthstatus*, *Healins*, *Med.delay.notget*, and *Mort.prob*. We expect the predictors in the *final model* to form a set in-between the sets of the predictors for the *reduced mode* and the *full model*. Now, the problem is how to find the *final model* using the *reduced* and *full models*. To this end, we carry out the following four steps.

In Step 1, we fit logistic regression with predictors in the *full model* and in the *reduced model*, respectively, to each of the five surrogate data sets for each of the 12 weeks.

In Step 2, the estimates and standard errors of the model coefficients for a given week are obtained using the algorithm described by Allison [12]. To be specific, let *M* = 5 be the number of surrogate data sets for the original incomplete data. Let *β*_*j*_ be the *j*th component of the model parameter vector *β*. For *k* = 1, …, *M*, let ${\hat{\beta}}_j^{(k)}$ denote the estimate of the model parameter *β*_*j*_ obtained from fitting the *k*th surrogate data set in a week and let ${S}_j^{(k)}$ be its associated standard error. Then the point estimate of *β*_*j*_ is given by the average of those estimates of *β*_*j*_ derived from the *M* imputed data sets:

4 $${\hat{\beta}}_j=\frac{1}{M}\sum_{k=1}^M{\hat{\beta}}_j^{(k)}.$$

To determine the variability associated with ${\hat{\beta}}_j$, one needs to incorporate both the within imputation variance, denoted *V*_*w*_, and the between imputation variance, denoted *V*_*b*_. According to Rubin’s rule [6], the total variance associated with the multiple imputation estimate ${\hat{\beta}}_j$ is given by $Var\left({\hat{\beta}}_j\right)={V}_w+\left(1+\frac{1}{M}\right){V}_b$, where ${V}_w=\frac{1}{M}\sum_{k=1}^M{\left\{{S}_j^{(k)}\right\}}^2$, and the between imputation variance, ${V}_b=\frac{1}{M-1}\sum_{k=1}^M{\left\{{\hat{\beta}}_j^{(k)}-{\hat{\beta}}_j\right\}}^2$, is inflated by a factor $\frac{1}{M}$ . As a result, the standard error associated with ${\hat{\beta}}_j$ is given by $se\left({\hat{\beta}}_j\right)=\sqrt{Var\left({\hat{\beta}}_j\right)}$, i.e.,

5 $$se\left({\hat{\beta}}_j\right)=\sqrt{\frac{1}{M}\sum_{k=1}^M{\left\{{S}_j^{(k)}\right\}}^2+\left(1+\frac{1}{M}\right)\left(\frac{1}{M-1}\right)\sum_{k=1}^M{\left\{{\hat{\beta}}_j^{(k)}-{\hat{\beta}}_j\right\}}^2}\kern0.5em .$$

We report in Tables S4 and S5 in the Supplementary Material the estimated results of the covariate effects obtained, respectively, from the *full* and *reduced models* for the data in 12 weeks, where the covariates marked with an asterisk are statistically significant with p-values smaller than 0.05 for more than 6 weeks. It is found that in addition to those covariates included in the reduced model, fitting the full model also shows that five additional covariates, *State*, *Rhispanic*, *Race*, *Numper*, and *Schoolenroll,* are statistically significant for more than 6 weeks’ data. Table S5 shows that almost all the covariates in the *reduced model* are statistically significant, with all the p-values derived from the data in 12 weeks smaller than 0.05.

Consequently, in Step 3, we take the 11 significant risk factors from the *reduced model*, and the 5 additional partially significant covariates suggested by fitting the *full model*, *State*, *Rhispanic*, *Race*, *Numper*, and *Schoolenroll,* to form the list of risk factors for the *final model.*

In Step 4, we construct the *final model *using the model form (1) to include the selected variables in Step 3 as predictors, where dummy variables are used to express categorical variables *State*, *Race*, *MS*, *Foodconf*, and *Hlthstatus* with levels more than two, yielding 28 variables in the model. The *final model* is then given by

6 $$\begin{aligned}\mathrm{logit}\ \pi =&{\beta}_0+{\beta}_1\times State. mild\\ &+{\beta}_2\times State. moderate. daily\\ &+{\beta}_3\times State. serious\\ &+{\beta}_4\times Age+{\beta}_5\times Male\\ &+{\beta}_6\times Rhispanic+{\beta}_7\times Race2\\ &+{\beta}_8\times Race3+{\beta}_9\times Race4\\ &+{\beta}_{10}\times MS2+{\beta}_{11}\times MS3\\ &+{\beta}_{12}\times MS4+{\beta}_{13}\times MS5\\ &+{\beta}_{14}\times Numper+{\beta}_{15}\times Numkid\\ &+{\beta}_{16}\times Wrkloss+{\beta}_{17}\times Anywork\\ &+{\beta}_{18}\times Foodconf2+{\beta}_{19}\times Foodconf3\\ &+{\beta}_{20}\times Foodconf4+{\beta}_{21}\times Hlthstatus2\\ &+{\beta}_{22}\times Hlthstatus3\\ &+{\beta}_{23}\times Hlthstatus4+{\beta}_{24}\times Hlthstatus5\\ &+{\beta}_{25}\times Healins\\ &+{\beta}_{26}\times Med. delay. notget+{\beta}_{27}\times Mort. prob\\ &+{\beta}_{28}\times Schoolenroll,\end{aligned}$$

where *β*_*j*_ is the regression coefficients for *j* = 0, 1, …, 28, and the subscript *i* is suppressed in *π* and the covariates for ease of exposition.

**Fig. 2:**
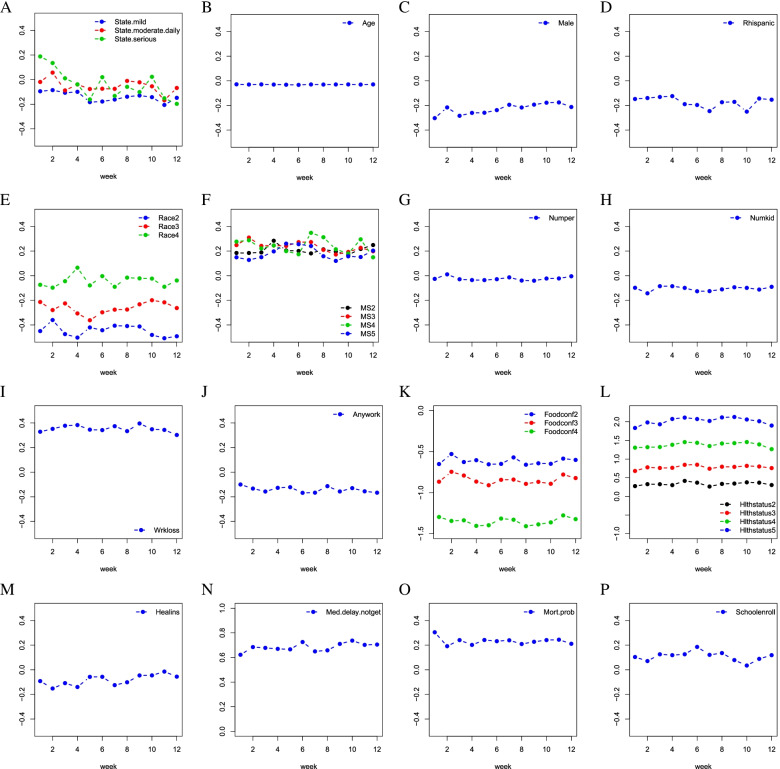
The estimates of the coefficients for all the factors in the final model are displayed against the week number

Then, we fit the final logistic model (6) to each of the imputed data sets for each of the 12 weeks; in the same manner as indicated by (4) and (5), we obtain the point estimates of the model parameters and the associated standard errors. To have a visual display, we plot in Fig. 2 the estimates of the coefficients for all the factors in the final model for 12 weeks; to precisely show the estimates, we report in Table 3 the point estimates for the covariate effects obtained from the *final model*, where we further calculate the average of the 12 estimates for each covariate and report the results in the last column. The associated standard errors and the p-values are deferred to Table S6 in the Supplementary Material. The results suggest that the factors *Numper, Healins* and *Schoolenroll* are only significant in some of 12 weeks, while other factors in the final models are significant in all 12 weeks.

**Table 3:** Results of the Final Model

Week	1	2	3	4	5	6	7	8	9	10	11	12	Average
Covariate													
(Intercept)	0.075	0.006	0.072	0.184	0.226	0.228	0.179	0.268	0.177	0.118	0.207	0.253	0.166
State.mild	-0.095	-0.085	-0.107	-0.099	-0.183	-0.178	-0.162	-0.139	-0.129	-0.143	-0.206	-0.148	-0.139
State.moderate.daily	-0.019	0.057	-0.088	-0.040	-0.076	-0.074	-0.075	-0.011	-0.022	-0.054	-0.168	-0.068	-0.053
State.serious	0.188	0.135	0.011	-0.038	-0.161	0.020	-0.133	-0.059	-0.101	0.023	-0.152	-0.197	-0.039
Age	-0.029	-0.030	-0.029	-0.031	-0.032	-0.033	-0.030	-0.030	-0.030	-0.029	-0.031	-0.029	-0.030
Male	-0.303	-0.216	-0.284	-0.261	-0.259	-0.238	-0.195	-0.217	-0.194	-0.178	-0.176	-0.213	-0.228
Rhispanic	-0.148	-0.140	-0.131	-0.124	-0.190	-0.196	-0.246	-0.174	-0.171	-0.250	-0.144	-0.154	-0.172
Race2	-0.450	-0.360	-0.474	-0.503	-0.420	-0.443	-0.406	-0.409	-0.412	-0.481	-0.508	-0.492	-0.446
Race3	-0.214	-0.279	-0.225	-0.306	-0.362	-0.297	-0.276	-0.275	-0.232	-0.199	-0.216	-0.263	-0.262
Race4	-0.074	-0.097	-0.045	0.065	-0.079	-0.004	-0.090	-0.016	-0.022	-0.024	-0.090	-0.039	-0.043
MS2	0.185	0.185	0.189	0.284	0.205	0.201	0.181	0.214	0.196	0.170	0.217	0.249	0.206
MS3	0.249	0.310	0.242	0.245	0.242	0.273	0.273	0.210	0.173	0.193	0.226	0.198	0.236
MS4	0.277	0.288	0.222	0.246	0.200	0.174	0.348	0.313	0.215	0.180	0.295	0.150	0.242
MS5	0.149	0.128	0.150	0.197	0.260	0.256	0.241	0.158	0.120	0.157	0.152	0.204	0.181
Numper	-0.026	0.011	-0.029	-0.035	-0.036	-0.028	-0.014	-0.039	-0.041	-0.023	-0.022	-0.005	-0.024
Numkid	-0.098	-0.143	-0.085	-0.085	-0.099	-0.126	-0.125	-0.111	-0.094	-0.099	-0.112	-0.090	-0.106
Wrkloss	0.328	0.352	0.377	0.383	0.345	0.342	0.373	0.333	0.395	0.349	0.343	0.302	0.352
Anywork	-0.100	-0.133	-0.157	-0.127	-0.122	-0.168	-0.167	-0.114	-0.157	-0.130	-0.156	-0.167	-0.141
Foodconf2	-0.651	-0.529	-0.627	-0.604	-0.654	-0.649	-0.570	-0.659	-0.642	-0.648	-0.585	-0.601	-0.618
Foodconf3	-0.866	-0.746	-0.791	-0.865	-0.909	-0.843	-0.840	-0.893	-0.868	-0.891	-0.779	-0.822	-0.843
Foodconf4	-1.296	-1.344	-1.337	-1.404	-1.397	-1.315	-1.329	-1.408	-1.386	-1.362	-1.277	-1.322	-1.348
Hlthstatus2	0.276	0.328	0.327	0.303	0.416	0.369	0.265	0.336	0.345	0.377	0.368	0.305	0.335
Hlthstatus3	0.682	0.779	0.762	0.767	0.847	0.849	0.742	0.796	0.791	0.818	0.802	0.759	0.783
Hlthstatus4	1.307	1.319	1.324	1.384	1.456	1.437	1.350	1.418	1.428	1.457	1.394	1.267	1.378
Hlthstatus5	1.834	1.983	1.933	2.076	2.112	2.074	2.022	2.117	2.129	2.062	2.015	1.899	2.021
Healins	-0.092	-0.152	-0.108	-0.140	-0.058	-0.057	-0.125	-0.101	-0.046	-0.045	-0.015	-0.056	-0.083
Med.delay.notget	0.622	0.685	0.678	0.670	0.666	0.726	0.650	0.658	0.710	0.737	0.702	0.705	0.684
Mort.prob	0.305	0.192	0.241	0.202	0.242	0.232	0.240	0.210	0.227	0.241	0.244	0.211	0.232
Schoolenroll	0.104	0.071	0.126	0.119	0.126	0.186	0.121	0.136	0.079	0.035	0.089	0.119	0.109

The table shows the point estimates of the covariate effects individually derived from the data in each of 12 weeks, together with their averages over 12 weeks shown in the last column

## Results

Figure 2 shows that the absolute values of coefficient estimates for some levels of variables *Foodconf* and *Hlthstatus* are greater than 1 (in Fig. 2K and L). The coefficient estimates of *Med.delay.notget* over 12 weeks are between 0.5 and 1 (in Fig. 2N). Other variables have coefficient estimates between -0.5 and 0.5.

To have an *overall* sense of the estimates of the predictor effects in the final model, we now utilize the *averages* reported in the last column of Table 3 to estimate the *relative change* in the odds of having mental issues with one unit increase in a predictor from its baseline while keeping other predictors unchanged, yet leaving the associated variability uncharacterized. Let ${\overline{\hat{\beta}}}_j$ represent the average of those estimates of the covariate effect *β*_*j*_ over the 12 weeks for *j* = 1, …, 28, which is a sensible estimate of *β*_*j*_, because the arithmetic average preserves the consistency if all the estimators obtained for the 12 weeks are consistent for *β*_*j*_. Using ${\overline{\hat{\beta}}}_j$ is advantageous in offering us a *single* estimate of *β*_*j*_ with generally expected smaller variability than those estimates obtained from each of the 12 weeks. If ${\overline{\hat{\beta}}}_j$ is negative, then $1-{\exp}\left({\overline{\hat{\beta}}}_j\right)$ shows an estimate of the *decrease* in the odds of having mental issues relative to the baseline; if ${\overline{\hat{\beta}}}_j$ is positive, then ${\exp}\left({\overline{\hat{\beta}}}_j\right)-1$ suggests an estimate of the *increase* in the odds of having mental issues relative to the baseline.

To be specific, for the variable *State* with *large daily increases of cases* as the baseline, people from *mild pandemic* States exhibit an estimate of 1 −  *exp* (−0.139) ≈ 13% decrease in the odds of having mental issues; people from the States with *moderate daily increases* show an estimate of 1 −  *exp* (−0.053) ≈ 5.16% degrease in the odds; people from serious pandemic States are generally associated with an estimate of 1 −  *exp* (−0.039) ≈ 3.82% decrease in the odds.

For *Age* and *Gender*, their averages of the estimates over the 12 weeks are -0.030 and -0.228, respectively, implying that one unit increase in *Age* is associated with about an estimate of 1 −  *exp* (−0.030) ≈ 2.96% decrease in the odds of occurrence of mental health problems; and being a male relative to a female is associated with an estimate of 1 −  *exp* (−0.228) ≈ 20.39% decrease in the odds of having mental health issues. Similarly, the 12-week estimated effects of *Rhispanic* indicate that the origin of Hispanic, Latino or Spanish is associated with a smaller odds of having mental issues than others. The 12-week mean of the coefficient estimates of *Rhispanic* is -0.172, leading to an estimate of the odds of mental health problem occurrence being reduced by around 1 −  *exp* (−0.172) ≈ 15.80%.

For the variable *Race* with *White* as the baseline, the 12-week mean of coefficient estimates for *Black* (Race2) and *Asian* (Race3) are -0.446 and -0.262, respectively, yielding an estimate of the odds of occurrence of mental health issues for *Black* and *Asian* to be, respectively, 1 −  *exp* (−0.446) ≈ 35.98% and 1 −  *exp* (−0.262) ≈ 23.05% less than *White*.

For *MS* (marital status) with *now married* as the baseline, an estimate of the increase in the odds of having mental issues relative to the baseline, is *exp*(0.206) − 1≈22.88%, *exp*(0.236) − 1≈26.62%, *exp*(0.242) − 1≈27.38%, and *exp*(0.181) − 1≈19.84%, respectively, for people who are *widowed* (MS2), *divorced* (MS3), *separated* (MS4)*,* or *never married* (MS5).

For predictors *Numper* and *Numkid*, the averages of the estimates suggest that the increase of the number of people and kids in the household is associated with the decrease of the odds of having mental issues. Specifically, one person increase in the household is associated with an estimate of 1 −  *exp* (−0.024)≈2.37% decrease in odds, and one more kid in the household is associated with an estimate of 1 −  *exp* (−0.106)≈10.06% decrease in the odds.

For the work-related factors *Wrkloss* and *Anywork*, the results shown in the last column in Table 3 indicate that experiencing a loss of employment income since March 13, 2020 is associated with an estimate of *exp*(0.352) − 1≈42.19% increase in the odds of having mental issues, and doing any work during the last 7 days is associated with an estimate of 1 −  *exp* (−0.141)≈13.15% decrease in the odds.

The 12-week results of *Foodconf* in Table 3 show that, with the *not at all confident* on the future food affordability as the baseline, an increase in the confidence of food affordability is negatively associated with the odds of having mental issues. On average of 12 weeks, shown in the last column in Table 3, the more confident in the food affordability, the less the odds of having mental issues. For example, the person who is very confident *(Foodconf4)* in the food affordability for the next four weeks demonstrates an estimate of 1 −  *exp* (−1.348)≈74.02% decrease in the odds of having mental issues, relative to the person who is *not at all confident*.

With *excellent* health conditions as the baseline, the estimates of *Hlthstatus* in Table 3 say that the worse the self-evaluated health condition, the larger the odds of having mental issues. Considering the worst level of health condition *poor* (Hlthstatus5) as an example, the average of the estimates over the 12 weeks yields that people in *poor* health conditions have an estimate of the odds of having mental issues *exp*(2.021)≈7.55 times higher than people of *excellent* health conditions. For other health-related predictors, *Healins* and *Med.delay.notget*, people who are currently covered by health insurance are associated with an estimate of 1 −  *exp* (−0.083)≈7.96% decrease in the odds of mental issues occurrence, and people who do not get medical care or have delayed medical care are associated with an estimate of *exp*(0.684) − 1≈98.18% increase in the odds.

For *Mort.prob* and *Schoolenroll*, people having rental or mortgage problems are associated with an estimate of *exp*(0.232) − 1≈26.15% increase in the odds of having mental health problems, and people whose household has kids enrolled in school are associated with an estimate of *exp*(0.109) − 1≈11.52% increase in the odds of having mental issues.

In summary, the factors in the *final model* associated with a *reduction* in the odds of having mental health issues include: States not having large daily increases of cases, older in age, being male, having a Hispanic, Latino or Spanish origin, being non-White, more people or kids in the household, having job during the last 7 days, having confidence in the food affordability in the future, and being covered by insurance. The factors in the *final model* associated with the *increase* in the odds of getting mental issues are: not married, experiencing loss of job, poor self-evaluations on health conditions, having problems in getting medical care and mortgage, and having kids enrolled in school.

## Discussion

In this paper we investigate the impact of the COVID-19 pandemic on the public mental health using an online survey data set from the United States. Prior to the analysis, we pre-process the data by combining some levels of certain variables in the hope to ameliorate the effects of the errors that are often induced in survey data, including recall bias, reporting error, uncertainty in providing a precise assessment of the situation, inability to decide a right scale to a question, and inconsistency in the answers to the same question that is phrased differently [15]. In addition, some variables are quite similar or even identical in nature, thus, combining them can help alleviate unwanted noise. Further, we employ multiple imputation to account for the missingness effects, and use the penalized logistic regression with the Lasso penalty to select important risk factors for mental health.

While this study offers us quantitative evidence how the COVID-19 pandemic can psychologically challenge the public, several limitations need to be pointed out. Firstly, the online survey data were designed to assess the pandemic impact from the social and economic perspectives, and they may not contain enough necessary factors related to mental health issues. In addition, the interaction effects between the predictors are not considered in our analysis, which may restrict the capacity of the model. Secondly, while the choice of *M* = 5 in our analysis follows the suggestion of Allison [12], it would be interesting to study how the variability may be incurred by setting different values for *M*.

Thirdly, though it is easy to see that the data exhibit arbitrary missingness patterns, or the so-called *intermittent missing data patterns*, it is difficult to tell what exactly the underlying missing data mechanism is, as in many other missing data problems [16]. Though the multiple imputation method is useful for handling missing data with the MAR mechanism [16], its performance can be considerably impacted by different proportions of missing values. Efforts of accounting for missingness effects do not always come to be rewarding. In the presence of excessive missing observations, the multiple imputation method, like any other method, can fail to yield sensible results even if the MAR mechanism is true. In such instances, one needs to be cautious to interpret the analysis results and be aware of potentially induced biases due to a high proportion of missing information.

Finally, in the analysis, we define the response variable to be binary by combining the information collected from four questions about mental health. While this approach gives a simple way to indicate the mental health status and is similarly taken by other authors (e.g., [10]), it is heuristic, as pointed out by a referee. It is thereby interesting to take the original four categorial variables as outcomes and conduct multivariate analysis to examine how those outcomes are associated with the covariates with missingness effects accommodated. Such analyses would be more sophisticated and require extra care to facilitate the association structures among the multiple response variables. Further, the yielded results may be less intuitive to interpret than those derived from using a single response variable.

## Conclusions

The analysis results unveil evidence-based findings to identify the groups who are psychologically vulnerable to the COVID-19 pandemic. This study provides helpful evidence to assist healthcare providers and policymakers to take steps for mitigating the pandemic effects on public mental health, especially in boosting public health care, improving public confidence in future food conditions, and creating more job opportunities.

## Supplementary Information

**Additional file 1.**

## Data Availability

The data sets analyzed here are available in the Bureau of the Census, Household Pulse Survey Public Use File (PUF) repository [4], *https://www.census.gov/programs-surveys/household-pulse-survey/datasets.html*.

## Abbreviations

COVID-19: Coronavirus disease 2019
MICE: multiple imputations by chained equations
Lasso: least absolute shrinkage and selection operator
