# Supplementary material for: COVID-19 impact on mental health
Source: BMC Med Res Methodol. 2022 Jan 14;22:15. doi: 10.1186/s12874-021-01411-w (PMC8758244; doi:10.1186/s12874-021-01411-w)
Supplement: Supplementary file 1 — Additional file 1. [file 12874_2021_1411_MOESM1_ESM.docx]

**Supplementary Material for**

**“COVID-19 Impact on Mental Health”**

by Jingyu Cui, Jingwei Lu, Yijia Weng, Grace Y. Yi, and Wenqing He

This supplementary material includes additional figures and tables for the data analyses that are discussed in the manuscript, Cui, J., Lu, J., Weng, Y., Yi, G. Y., and He, W. (2021). COVID-19 Impact on Mental Health over Time. *BMC Medical Research Methodology.*

| 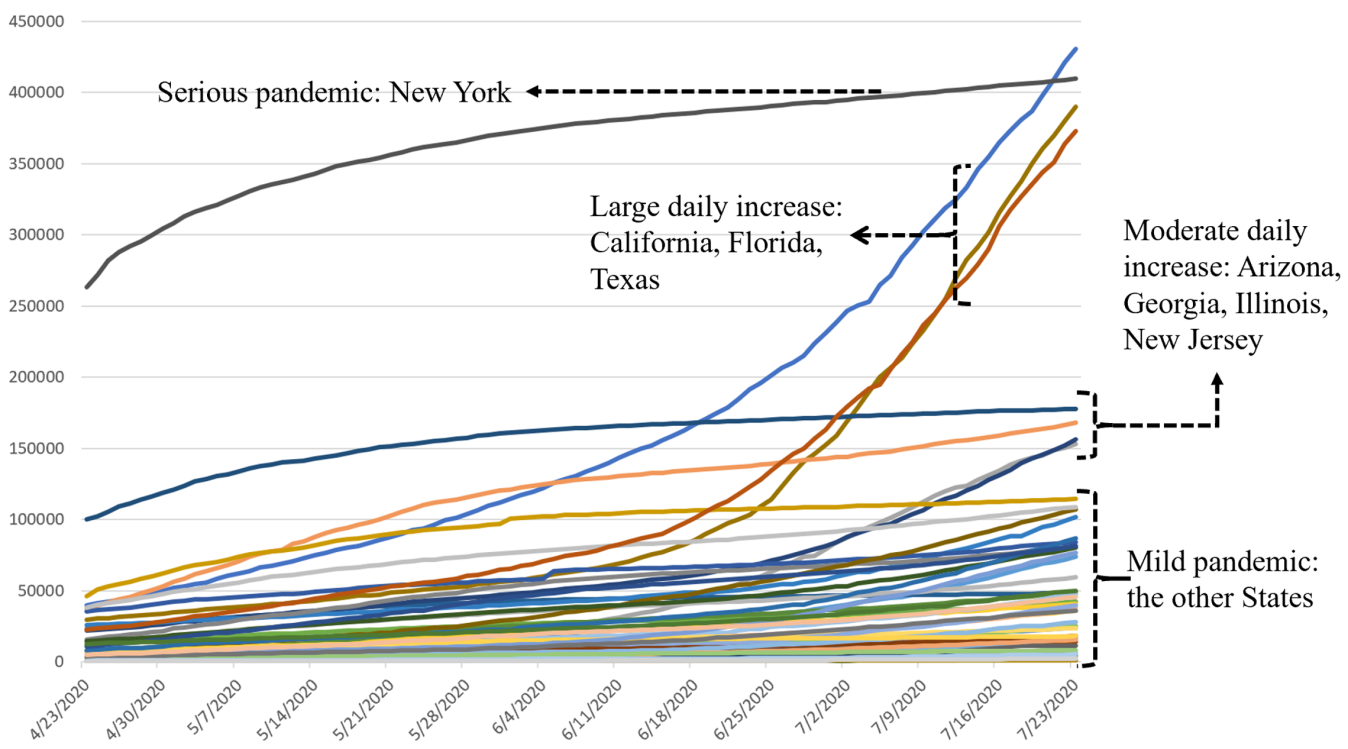  **Figure S1:** The curves of cumulative confirmed cases for all the US states between April 23, 2020 and July 23, 2020, derived from the data from the *Centers for Disease Control and Prevention* (https://data.cdc.gov/). The four categories of the severity of the pandemic are also shown. The State of New York, with a cumulative cases line on the very top, is defined as the *serious* pandemic state. The states with stable cumulative cases staying at the bottom are regarded as the *mild* pandemic states. In-between, the states (California, Florida, and Texas) with steep curves are taken as the states with *large daily increases*, and the states (Arizona, Georgia, Illinois, and New Jersey) with less steep curves are regarded as the states with *moderate daily increases*. |
| --- |

| **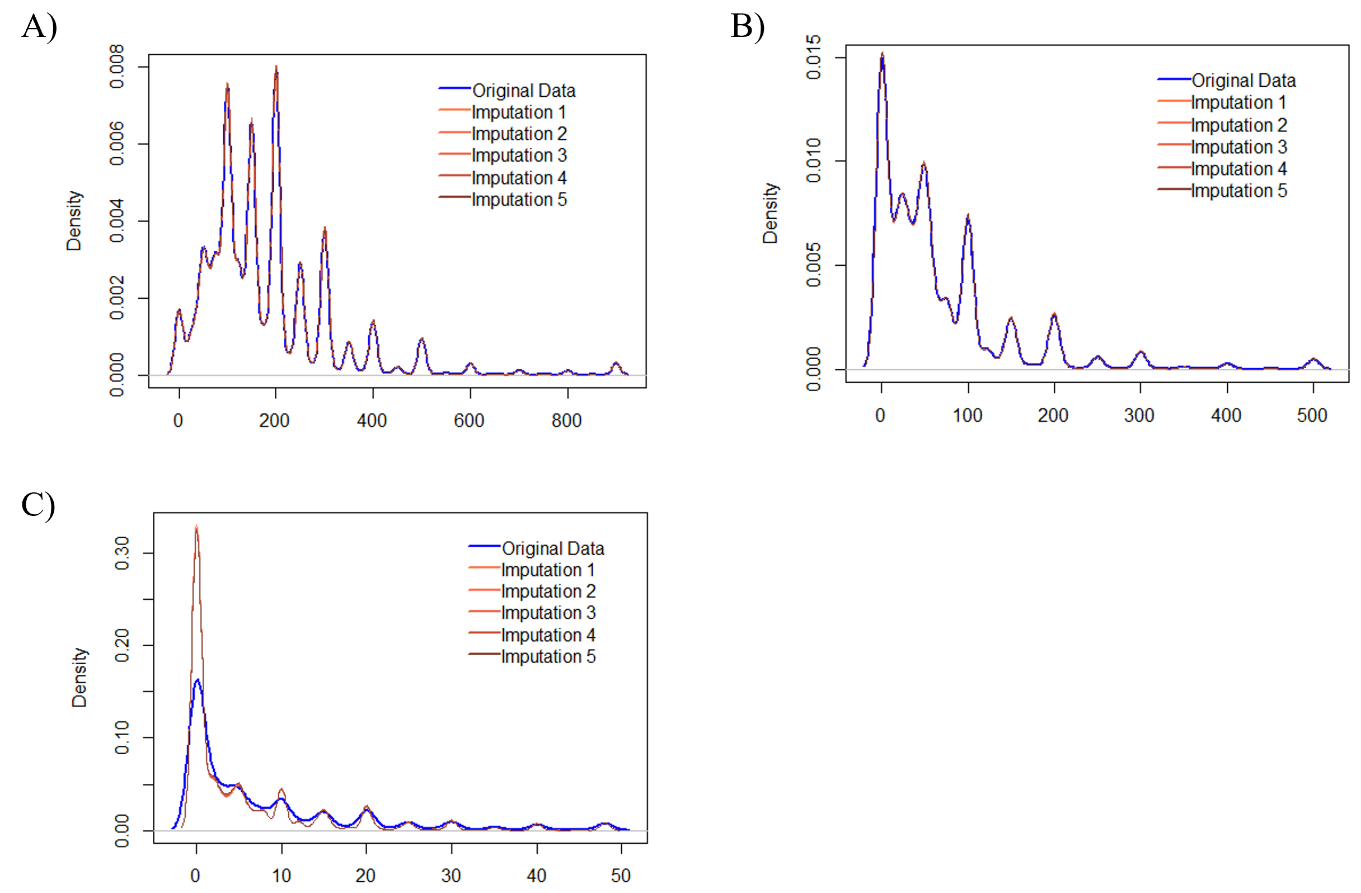** |
| --- |
| **Figure S2**: Estimated density curves of the observed data in week 6 and their five imputed data for the three continuous variables: A) *Tspndfood*, B) *Tspndprpd,* and C) *Ttch_Hrs*. In each plot, the blue curve denotes the density of the variable in the original data, and five red curves (from light coral red to dark coral red) represent the densities of the variable in the 5 imputed data sets; those curves are obtained using the R function *density*. |

| Table S1. Proportions of Two Levels for *Anywork* in Week 6 That Are Obtained for the Original Data and the Five Imputed Data Sets. | | |
| --- | --- | --- |
| Level  Data type | Yes | No |
| Original Data | 0.578 | 0.422 |
| Imputation 1 | 0.577 | 0.423 |
| Imputation 2 | 0.577 | 0.423 |
| Imputation 3 | 0.577 | 0.423 |
| Imputation 4 | 0.577 | 0.423 |
| Imputation 5 | 0.577 | 0.423 |
| *Anywork* is an indicator variable, indicating if the participant did any work for either pay or profit in the last 7 days. | | |

| Table S2. Proportions of Different Levels for *Kindwork* in Week 6 That Are Obtained for the Original Data and the Five Imputed Data Sets. | | | | | |
| --- | --- | --- | --- | --- | --- |
|  |  |  |  |  |  |
| Level  Data type | Government | Private Company | Non-profit  Organization | Self-employed | Family Business |
| Original Data | 0.179 | 0.549 | 0.133 | 0.116 | 0.023 |
| Imputation 1 | 0.170 | 0.522 | 0.131 | 0.149 | 0.028 |
| Imputation 2 | 0.169 | 0.527 | 0.131 | 0.145 | 0.028 |
| Imputation 3 | 0.172 | 0.522 | 0.133 | 0.145 | 0.027 |
| Imputation 4 | 0.169 | 0.525 | 0.131 | 0.148 | 0.027 |
| Imputation 5 | 0.170 | 0.525 | 0.131 | 0.144 | 0.029 |
| *Kindwork* is a categorical variable with five levels, 1: government; 2: private company; 3: non-profit organization including tax and charitable organizations; 4: self-employed; 5: working in a family business, indicating the kind of work of the participant. | | | | | |

| Table S3. Proportions of Different Levels for *Income* in Week 6 That Are Obtained for the Original Data and the Imputed Data Sets. | | | | | | | | | | | | | | | | |
| --- | --- | --- | --- | --- | --- | --- | --- | --- | --- | --- | --- | --- | --- | --- | --- | --- |
|  |  |  | |  | |  | |  | |  | |  | |  | | |
| Level  Data type | <$25000 | | $25000 - 34999 | | $35000 - 49999 | | $50000 - 74999 | | $75000 - 99999 | | $100000 - 149999 | | $150000 - 199999 | | $\geq$$200000 |  |
| Original Data | 0.104 | | 0.088 | | 0.109 | | 0.177 | | 0.148 | | 0.186 | | 0.090 | | 0.099 |  |
| Imputation 1 | 0.107 | | 0.089 | | 0.110 | | 0.178 | | 0.149 | | 0.183 | | 0.088 | | 0.096 |  |
| Imputation 2 | 0.108 | | 0.089 | | 0.111 | | 0.178 | | 0.148 | | 0.183 | | 0.088 | | 0.096 |  |
| Imputation 3 | 0.107 | | 0.090 | | 0.110 | | 0.177 | | 0.148 | | 0.184 | | 0.088 | | 0.096 |  |
| Imputation 4 | 0.108 | | 0.090 | | 0.110 | | 0.177 | | 0.147 | | 0.184 | | 0.088 | | 0.096 |  |
| Imputation 5 | 0.108 | | 0.089 | | 0.109 | | 0.177 | | 0.148 | | 0.184 | | 0.088 | | 0.096 |  |
| *Income* is a categorical variable with eight levels, 1: less than $25,000; 2: $25,000 - $34,999; 3: $35,000 - $49,999; 4: $50,000 - $74,999; 5: $75,000 - $99,999; 6: $100,000 - $149,999; 7: $150,000 - $199,999; 8: $200,000 and above, indicating what the total household income was before taxes. | | | | | | | | | | | | | | | | |

**Table S4.** Full Model: Point Estimates, Standard Errors and P-values of the Covariate Effects Individually Derived from the Data in Weeks 1-3

| Week  Covariate |  | 1 |  |  | 2 |  |  | 3 |  |
| --- | --- | --- | --- | --- | --- | --- | --- | --- | --- |
|  | Estimate | s.e. | p-value | Estimate | s.e. | p-value | Estimate | s.e. | p-value |
| (Intercept) | -0.301 | 0.189 | 0.111 | 0.020 | 0.281 | 0.943 | 0.069 | 0.129 | 0.593 |
| State.mild* | -0.070 | 0.032 | 0.028 | -0.066 | 0.044 | 0.133 | -0.092 | 0.023 | 0.000 |
| State.moderate.daily | -0.009 | 0.046 | 0.838 | 0.060 | 0.065 | 0.363 | -0.083 | 0.035 | 0.017 |
| State.serious | 0.177 | 0.074 | 0.017 | 0.121 | 0.103 | 0.241 | 0.000 | 0.058 | 0.999 |
| Age* | -0.029 | 0.001 | 0.000 | -0.031 | 0.001 | 0.000 | -0.029 | 0.001 | 0.000 |
| Male* | -0.317 | 0.026 | 0.000 | -0.229 | 0.033 | 0.000 | -0.285 | 0.020 | 0.000 |
| Rhispanic* | -0.138 | 0.047 | 0.004 | -0.120 | 0.059 | 0.044 | -0.124 | 0.033 | 0.000 |
| Race2* | -0.440 | 0.040 | 0.000 | -0.341 | 0.060 | 0.000 | -0.479 | 0.032 | 0.000 |
| Race3* | -0.247 | 0.066 | 0.000 | -0.292 | 0.080 | 0.000 | -0.252 | 0.046 | 0.000 |
| Race4 | -0.063 | 0.050 | 0.211 | -0.079 | 0.068 | 0.245 | -0.040 | 0.044 | 0.373 |
| Educ2 | 0.151 | 0.158 | 0.341 | -0.158 | 0.230 | 0.490 | -0.073 | 0.108 | 0.503 |
| Educ3 | 0.252 | 0.162 | 0.119 | -0.101 | 0.229 | 0.661 | 0.041 | 0.107 | 0.702 |
| Educ4 | 0.339 | 0.162 | 0.036 | -0.027 | 0.231 | 0.907 | 0.082 | 0.112 | 0.468 |
| MS2* | 0.227 | 0.063 | 0.000 | 0.229 | 0.081 | 0.005 | 0.221 | 0.055 | 0.000 |
| MS3* | 0.285 | 0.034 | 0.000 | 0.355 | 0.046 | 0.000 | 0.263 | 0.028 | 0.000 |
| MS4* | 0.309 | 0.072 | 0.000 | 0.331 | 0.108 | 0.002 | 0.242 | 0.061 | 0.000 |
| MS5* | 0.181 | 0.033 | 0.000 | 0.159 | 0.047 | 0.001 | 0.170 | 0.028 | 0.000 |
| Numper* | -0.027 | 0.012 | 0.031 | 0.012 | 0.016 | 0.461 | -0.028 | 0.009 | 0.001 |
| Numkid* | -0.110 | 0.022 | 0.000 | -0.165 | 0.024 | 0.000 | -0.097 | 0.015 | 0.000 |
| Income2 | -0.054 | 0.044 | 0.224 | 0.071 | 0.061 | 0.248 | -0.091 | 0.040 | 0.023 |
| Income3 | -0.043 | 0.049 | 0.380 | 0.018 | 0.063 | 0.777 | 0.003 | 0.040 | 0.931 |
| Income4 | -0.032 | 0.043 | 0.466 | 0.046 | 0.056 | 0.403 | 0.052 | 0.035 | 0.134 |
| Income5 | 0.064 | 0.049 | 0.189 | 0.059 | 0.064 | 0.357 | -0.016 | 0.037 | 0.672 |
| Income6 | 0.044 | 0.050 | 0.372 | 0.089 | 0.063 | 0.160 | 0.003 | 0.041 | 0.950 |
| Income7 | 0.035 | 0.067 | 0.602 | 0.059 | 0.084 | 0.484 | 0.030 | 0.046 | 0.523 |
| Income8 | 0.041 | 0.064 | 0.520 | 0.094 | 0.088 | 0.285 | 0.021 | 0.048 | 0.671 |
| Wrkloss* | 0.314 | 0.026 | 0.000 | 0.344 | 0.035 | 0.000 | 0.373 | 0.020 | 0.000 |
| Anywork* | -0.126 | 0.025 | 0.000 | -0.152 | 0.034 | 0.000 | -0.178 | 0.021 | 0.000 |
| Kindwork2 | 0.124 | 0.039 | 0.002 | 0.057 | 0.050 | 0.256 | 0.034 | 0.027 | 0.206 |
| Kindwork3 | 0.148 | 0.049 | 0.003 | 0.135 | 0.077 | 0.081 | 0.060 | 0.038 | 0.113 |
| Kindwork4 | 0.209 | 0.058 | 0.000 | 0.050 | 0.064 | 0.429 | 0.052 | 0.035 | 0.135 |
| Kindwork5 | 0.246 | 0.088 | 0.005 | 0.303 | 0.130 | 0.020 | 0.027 | 0.122 | 0.824 |
| Emppay | 0.028 | 0.045 | 0.532 | -0.239 | 0.114 | 0.035 | 0.034 | 0.047 | 0.480 |
| Foodcon.change | -0.042 | 0.055 | 0.446 | -0.068 | 0.072 | 0.346 | -0.087 | 0.044 | 0.048 |
| Freefood | -0.032 | 0.043 | 0.461 | -0.037 | 0.062 | 0.546 | -0.029 | 0.033 | 0.373 |
| Tspndfood | 0.000 | 0.000 | 0.309 | 0.000 | 0.000 | 0.085 | 0.000 | 0.000 | 0.171 |
| Tspndprpd | 0.000 | 0.000 | 0.054 | 0.000 | 0.000 | 0.591 | 0.000 | 0.000 | 0.267 |
| Foodconf2* | -0.653 | 0.043 | 0.000 | -0.535 | 0.058 | 0.000 | -0.629 | 0.033 | 0.000 |
| Foodconf3* | -0.879 | 0.048 | 0.000 | -0.762 | 0.064 | 0.000 | -0.801 | 0.038 | 0.000 |
| Foodconf4* | -1.336 | 0.050 | 0.000 | -1.379 | 0.070 | 0.000 | -1.365 | 0.038 | 0.000 |
| Hlthstatus2* | 0.297 | 0.037 | 0.000 | 0.342 | 0.049 | 0.000 | 0.338 | 0.028 | 0.000 |
| Hlthstatus3* | 0.727 | 0.036 | 0.000 | 0.809 | 0.051 | 0.000 | 0.786 | 0.029 | 0.000 |
| Hlthstatus4* | 1.356 | 0.043 | 0.000 | 1.359 | 0.058 | 0.000 | 1.359 | 0.034 | 0.000 |
| Hlthstatus5* | 1.881 | 0.067 | 0.000 | 2.036 | 0.093 | 0.000 | 1.973 | 0.051 | 0.000 |
| Healins* | -0.101 | 0.042 | 0.017 | -0.149 | 0.060 | 0.012 | -0.125 | 0.037 | 0.001 |
| Med.delay.notget* | 0.603 | 0.023 | 0.000 | 0.662 | 0.031 | 0.000 | 0.662 | 0.019 | 0.000 |
| Mort.prob* | 0.313 | 0.034 | 0.000 | 0.193 | 0.042 | 0.000 | 0.253 | 0.037 | 0.000 |
| Schoolenroll* | 0.106 | 0.045 | 0.017 | 0.079 | 0.058 | 0.173 | 0.130 | 0.030 | 0.000 |
| Ttch_Hrs | 0.005 | 0.002 | 0.005 | 0.007 | 0.002 | 0.000 | 0.004 | 0.001 | 0.000 |
| * indicates the predictors are considered to be statistically significant in the sense that their p-values are smaller than 0.05 for more than 6 weeks. | | | | | | | | | |

**Table S4 (continued).** Full Model: Point Estimates, Standard Errors and P-values of the Covariate Effects Individually Derived from the Data in Weeks 4-6

| Week  Covariate |  | 4 |  |  | 5 |  |  | 6 |  |
| --- | --- | --- | --- | --- | --- | --- | --- | --- | --- |
|  | Estimate | s.e. | p-value | Estimate | s.e. | p-value | Estimate | s.e. | p-value |
| (Intercept) | -0.008 | 0.193 | 0.969 | 0.011 | 0.148 | 0.942 | 0.055 | 0.167 | 0.741 |
| State.mild* | -0.079 | 0.027 | 0.004 | -0.174 | 0.028 | 0.000 | -0.167 | 0.030 | 0.000 |
| State.moderate.daily | -0.027 | 0.041 | 0.510 | -0.074 | 0.043 | 0.088 | -0.071 | 0.043 | 0.097 |
| State.serious | -0.039 | 0.068 | 0.566 | -0.169 | 0.067 | 0.012 | -0.010 | 0.076 | 0.891 |
| Age* | -0.031 | 0.001 | 0.000 | -0.033 | 0.001 | 0.000 | -0.034 | 0.001 | 0.000 |
| Male* | -0.267 | 0.022 | 0.000 | -0.259 | 0.022 | 0.000 | -0.234 | 0.023 | 0.000 |
| Rhispanic* | -0.123 | 0.037 | 0.001 | -0.173 | 0.034 | 0.000 | -0.168 | 0.039 | 0.000 |
| Race2* | -0.506 | 0.039 | 0.000 | -0.407 | 0.037 | 0.000 | -0.421 | 0.040 | 0.000 |
| Race3* | -0.326 | 0.052 | 0.000 | -0.382 | 0.049 | 0.000 | -0.320 | 0.060 | 0.000 |
| Race4 | 0.064 | 0.046 | 0.165 | -0.073 | 0.051 | 0.151 | 0.016 | 0.052 | 0.752 |
| Educ2 | 0.004 | 0.139 | 0.976 | 0.138 | 0.116 | 0.234 | 0.148 | 0.144 | 0.304 |
| Educ3 | 0.110 | 0.138 | 0.424 | 0.218 | 0.116 | 0.060 | 0.279 | 0.146 | 0.056 |
| Educ4 | 0.114 | 0.140 | 0.415 | 0.263 | 0.120 | 0.029 | 0.338 | 0.150 | 0.024 |
| MS2* | 0.299 | 0.052 | 0.000 | 0.235 | 0.052 | 0.000 | 0.238 | 0.056 | 0.000 |
| MS3* | 0.256 | 0.030 | 0.000 | 0.266 | 0.031 | 0.000 | 0.299 | 0.033 | 0.000 |
| MS4* | 0.258 | 0.067 | 0.000 | 0.227 | 0.066 | 0.001 | 0.205 | 0.071 | 0.004 |
| MS5* | 0.204 | 0.030 | 0.000 | 0.278 | 0.029 | 0.000 | 0.267 | 0.034 | 0.000 |
| Numper* | -0.035 | 0.009 | 0.000 | -0.033 | 0.010 | 0.001 | -0.019 | 0.010 | 0.067 |
| Numkid* | -0.100 | 0.016 | 0.000 | -0.102 | 0.018 | 0.000 | -0.134 | 0.019 | 0.000 |
| Income2 | -0.005 | 0.040 | 0.910 | -0.053 | 0.043 | 0.219 | -0.084 | 0.048 | 0.077 |
| Income3 | 0.042 | 0.039 | 0.286 | 0.009 | 0.046 | 0.838 | 0.031 | 0.044 | 0.488 |
| Income4 | -0.005 | 0.041 | 0.912 | -0.004 | 0.039 | 0.929 | 0.046 | 0.039 | 0.239 |
| Income5 | -0.052 | 0.043 | 0.225 | 0.039 | 0.051 | 0.440 | -0.004 | 0.048 | 0.934 |
| Income6 | -0.002 | 0.043 | 0.958 | 0.016 | 0.052 | 0.759 | 0.026 | 0.050 | 0.598 |
| Income7 | 0.010 | 0.058 | 0.867 | 0.047 | 0.056 | 0.395 | 0.049 | 0.060 | 0.415 |
| Income8 | -0.038 | 0.055 | 0.493 | 0.087 | 0.059 | 0.144 | 0.047 | 0.058 | 0.422 |
| Wrkloss* | 0.379 | 0.021 | 0.000 | 0.341 | 0.022 | 0.000 | 0.330 | 0.024 | 0.000 |
| Anywork* | -0.135 | 0.023 | 0.000 | -0.137 | 0.023 | 0.000 | -0.184 | 0.026 | 0.000 |
| Kindwork2 | 0.000 | 0.035 | 0.995 | 0.013 | 0.032 | 0.680 | 0.045 | 0.039 | 0.251 |
| Kindwork3 | -0.024 | 0.042 | 0.578 | 0.029 | 0.049 | 0.555 | 0.087 | 0.078 | 0.266 |
| Kindwork4 | 0.003 | 0.041 | 0.948 | 0.051 | 0.042 | 0.221 | 0.100 | 0.060 | 0.097 |
| Kindwork5 | 0.064 | 0.079 | 0.419 | 0.132 | 0.089 | 0.136 | 0.260 | 0.073 | 0.000 |
| Emppay | -0.094 | 0.037 | 0.012 | -0.071 | 0.050 | 0.152 | -0.130 | 0.035 | 0.000 |
| Foodcon.change | 0.054 | 0.046 | 0.242 | 0.021 | 0.046 | 0.644 | -0.095 | 0.047 | 0.043 |
| Freefood | 0.013 | 0.038 | 0.728 | 0.015 | 0.040 | 0.705 | -0.036 | 0.046 | 0.435 |
| Tspndfood | 0.000 | 0.000 | 0.461 | 0.000 | 0.000 | 0.995 | 0.000 | 0.000 | 0.156 |
| Tspndprpd | 0.000 | 0.000 | 0.017 | 0.000 | 0.000 | 0.962 | 0.000 | 0.000 | 0.331 |
| Foodconf2* | -0.601 | 0.044 | 0.000 | -0.659 | 0.037 | 0.000 | -0.646 | 0.043 | 0.000 |
| Foodconf3* | -0.865 | 0.042 | 0.000 | -0.924 | 0.040 | 0.000 | -0.850 | 0.048 | 0.000 |
| Foodconf4* | -1.412 | 0.044 | 0.000 | -1.437 | 0.042 | 0.000 | -1.348 | 0.046 | 0.000 |
| Hlthstatus2* | 0.308 | 0.037 | 0.000 | 0.430 | 0.033 | 0.000 | 0.382 | 0.036 | 0.000 |
| Hlthstatus3* | 0.785 | 0.033 | 0.000 | 0.876 | 0.033 | 0.000 | 0.885 | 0.038 | 0.000 |
| Hlthstatus4* | 1.406 | 0.038 | 0.000 | 1.497 | 0.038 | 0.000 | 1.484 | 0.044 | 0.000 |
| Hlthstatus5* | 2.103 | 0.057 | 0.000 | 2.156 | 0.060 | 0.000 | 2.129 | 0.064 | 0.000 |
| Healins* | -0.143 | 0.044 | 0.001 | -0.060 | 0.035 | 0.085 | -0.059 | 0.042 | 0.164 |
| Med.delay.notget* | 0.658 | 0.021 | 0.000 | 0.653 | 0.021 | 0.000 | 0.707 | 0.023 | 0.000 |
| Mort.prob* | 0.207 | 0.029 | 0.000 | 0.249 | 0.033 | 0.000 | 0.246 | 0.034 | 0.000 |
| Schoolenroll* | 0.119 | 0.041 | 0.004 | 0.130 | 0.032 | 0.000 | 0.199 | 0.043 | 0.000 |
| Ttch_Hrs | 0.006 | 0.003 | 0.050 | 0.000 | 0.001 | 0.968 | 0.003 | 0.002 | 0.080 |
| * indicates the predictors are considered to be statistically significant in the sense that their p-values are smaller than 0.05 for more than 6 weeks. | | | | | | | | | |

**Table S4 (continued).** Full Model: Point Estimates, Standard Errors and P-values of the Covariate Effects Individually Derived from the Data in Weeks 7-9

| Week  Covariate |  | 7 |  |  | 8 |  |  | 9 |  |
| --- | --- | --- | --- | --- | --- | --- | --- | --- | --- |
|  | Estimate | s.e. | p-value | Estimate | s.e. | p-value | Estimate | s.e. | p-value |
| (Intercept) | -0.084 | 0.181 | 0.643 | -0.038 | 0.159 | 0.811 | 0.194 | 0.162 | 0.231 |
| State.mild* | -0.151 | 0.030 | 0.000 | -0.130 | 0.026 | 0.000 | -0.120 | 0.025 | 0.000 |
| State.moderate.daily | -0.072 | 0.043 | 0.096 | -0.010 | 0.036 | 0.786 | -0.022 | 0.038 | 0.567 |
| State.serious | -0.167 | 0.077 | 0.031 | -0.092 | 0.067 | 0.172 | -0.115 | 0.067 | 0.087 |
| Age* | -0.031 | 0.001 | 0.000 | -0.031 | 0.001 | 0.000 | -0.030 | 0.001 | 0.000 |
| Male* | -0.199 | 0.025 | 0.000 | -0.225 | 0.020 | 0.000 | -0.196 | 0.021 | 0.000 |
| Rhispanic* | -0.223 | 0.043 | 0.000 | -0.158 | 0.035 | 0.000 | -0.157 | 0.037 | 0.000 |
| Race2* | -0.390 | 0.042 | 0.000 | -0.409 | 0.033 | 0.000 | -0.413 | 0.038 | 0.000 |
| Race3* | -0.288 | 0.056 | 0.000 | -0.300 | 0.046 | 0.000 | -0.259 | 0.050 | 0.000 |
| Race4 | -0.083 | 0.053 | 0.119 | -0.011 | 0.040 | 0.792 | -0.017 | 0.040 | 0.664 |
| Educ2 | 0.214 | 0.162 | 0.187 | 0.201 | 0.120 | 0.093 | 0.009 | 0.126 | 0.943 |
| Educ3 | 0.265 | 0.159 | 0.096 | 0.289 | 0.120 | 0.016 | 0.091 | 0.126 | 0.472 |
| Educ4 | 0.318 | 0.159 | 0.046 | 0.313 | 0.122 | 0.010 | 0.130 | 0.127 | 0.306 |
| MS2* | 0.213 | 0.058 | 0.000 | 0.251 | 0.049 | 0.000 | 0.234 | 0.051 | 0.000 |
| MS3* | 0.302 | 0.034 | 0.000 | 0.242 | 0.029 | 0.000 | 0.203 | 0.030 | 0.000 |
| MS4* | 0.379 | 0.076 | 0.000 | 0.348 | 0.060 | 0.000 | 0.251 | 0.062 | 0.000 |
| MS5* | 0.258 | 0.034 | 0.000 | 0.185 | 0.027 | 0.000 | 0.147 | 0.027 | 0.000 |
| Numper* | -0.012 | 0.010 | 0.253 | -0.037 | 0.009 | 0.000 | -0.042 | 0.010 | 0.000 |
| Numkid* | -0.136 | 0.020 | 0.000 | -0.117 | 0.014 | 0.000 | -0.094 | 0.015 | 0.000 |
| Income2 | -0.018 | 0.050 | 0.719 | -0.006 | 0.037 | 0.879 | -0.010 | 0.038 | 0.796 |
| Income3 | 0.025 | 0.051 | 0.630 | 0.024 | 0.037 | 0.520 | -0.009 | 0.039 | 0.808 |
| Income4 | 0.054 | 0.049 | 0.266 | 0.078 | 0.034 | 0.022 | 0.052 | 0.036 | 0.158 |
| Income5 | 0.040 | 0.049 | 0.420 | 0.075 | 0.041 | 0.068 | 0.069 | 0.041 | 0.091 |
| Income6 | 0.043 | 0.050 | 0.394 | 0.081 | 0.040 | 0.042 | 0.053 | 0.041 | 0.195 |
| Income7 | 0.028 | 0.062 | 0.658 | 0.090 | 0.052 | 0.083 | 0.081 | 0.050 | 0.107 |
| Income8 | 0.168 | 0.062 | 0.007 | 0.050 | 0.053 | 0.348 | 0.057 | 0.053 | 0.277 |
| Wrkloss* | 0.365 | 0.026 | 0.000 | 0.326 | 0.022 | 0.000 | 0.398 | 0.023 | 0.000 |
| Anywork* | -0.176 | 0.027 | 0.000 | -0.128 | 0.020 | 0.000 | -0.172 | 0.021 | 0.000 |
| Kindwork2 | 0.016 | 0.042 | 0.701 | 0.009 | 0.037 | 0.811 | -0.062 | 0.030 | 0.041 |
| Kindwork3 | 0.053 | 0.048 | 0.263 | 0.021 | 0.052 | 0.689 | -0.054 | 0.060 | 0.367 |
| Kindwork4 | 0.024 | 0.050 | 0.636 | 0.066 | 0.040 | 0.095 | -0.076 | 0.037 | 0.043 |
| Kindwork5 | 0.059 | 0.105 | 0.576 | 0.079 | 0.111 | 0.474 | 0.088 | 0.087 | 0.313 |
| Emppay | -0.150 | 0.058 | 0.010 | -0.001 | 0.033 | 0.988 | -0.053 | 0.052 | 0.308 |
| Foodcon.change | -0.038 | 0.057 | 0.506 | -0.016 | 0.045 | 0.725 | -0.056 | 0.047 | 0.232 |
| Freefood | 0.029 | 0.042 | 0.495 | -0.023 | 0.038 | 0.545 | -0.083 | 0.037 | 0.026 |
| Tspndfood | 0.000 | 0.000 | 0.083 | 0.000 | 0.000 | 0.160 | 0.000 | 0.000 | 0.112 |
| Tspndprpd | 0.000 | 0.000 | 0.007 | 0.000 | 0.000 | 0.214 | 0.000 | 0.000 | 0.603 |
| Foodconf2* | -0.570 | 0.050 | 0.000 | -0.667 | 0.035 | 0.000 | -0.647 | 0.035 | 0.000 |
| Foodconf3* | -0.846 | 0.050 | 0.000 | -0.912 | 0.038 | 0.000 | -0.882 | 0.038 | 0.000 |
| Foodconf4* | -1.359 | 0.053 | 0.000 | -1.445 | 0.040 | 0.000 | -1.427 | 0.042 | 0.000 |
| Hlthstatus2* | 0.284 | 0.037 | 0.000 | 0.344 | 0.034 | 0.000 | 0.354 | 0.033 | 0.000 |
| Hlthstatus3* | 0.776 | 0.038 | 0.000 | 0.819 | 0.032 | 0.000 | 0.815 | 0.033 | 0.000 |
| Hlthstatus4* | 1.396 | 0.043 | 0.000 | 1.456 | 0.036 | 0.000 | 1.466 | 0.038 | 0.000 |
| Hlthstatus5* | 2.078 | 0.067 | 0.000 | 2.163 | 0.055 | 0.000 | 2.178 | 0.059 | 0.000 |
| Healins* | -0.126 | 0.044 | 0.004 | -0.116 | 0.036 | 0.001 | -0.067 | 0.036 | 0.061 |
| Med.delay.notget* | 0.629 | 0.025 | 0.000 | 0.639 | 0.019 | 0.000 | 0.695 | 0.021 | 0.000 |
| Mort.prob* | 0.246 | 0.039 | 0.000 | 0.219 | 0.028 | 0.000 | 0.239 | 0.032 | 0.000 |
| Schoolenroll* | 0.122 | 0.059 | 0.038 | 0.135 | 0.023 | 0.000 | 0.079 | 0.037 | 0.032 |
| Ttch_Hrs | 0.004 | 0.002 | 0.071 | 0.005 | 0.002 | 0.004 | 0.001 | 0.003 | 0.638 |
| * indicates the predictors are considered to be statistically significant in the sense that their p-values are smaller than 0.05 for more than 6 weeks. | | | | | | | | | |

**Table S4 (continued).** Full Model: Point Estimates, Standard Errors and P-values of the Covariate Effects Individually Derived from the Data in Weeks 10-12

| Week  Covariate |  | 10 | |  |  | 11 |  |  | 12 |  |
| --- | --- | --- | --- | --- | --- | --- | --- | --- | --- | --- |
|  | Estimate | | s.e. | p-value | Estimate | s.e. | p-value | Estimate | s.e. | p-value |
| (Intercept) | -0.130 | | 0.180 | 0.472 | 0.002 | 0.148 | 0.990 | 0.189 | 0.147 | 0.200 |
| State.mild* | -0.132 | | 0.028 | 0.000 | -0.196 | 0.026 | 0.000 | -0.139 | 0.028 | 0.000 |
| State.moderate.daily | -0.047 | | 0.039 | 0.229 | -0.166 | 0.040 | 0.000 | -0.065 | 0.040 | 0.099 |
| State.serious | 0.021 | | 0.069 | 0.762 | -0.157 | 0.068 | 0.021 | -0.209 | 0.070 | 0.003 |
| Age* | -0.030 | | 0.001 | 0.000 | -0.031 | 0.001 | 0.000 | -0.030 | 0.001 | 0.000 |
| Male* | -0.185 | | 0.022 | 0.000 | -0.177 | 0.021 | 0.000 | -0.210 | 0.023 | 0.000 |
| Rhispanic* | -0.242 | | 0.038 | 0.000 | -0.136 | 0.036 | 0.000 | -0.137 | 0.035 | 0.000 |
| Race2* | -0.478 | | 0.041 | 0.000 | -0.506 | 0.038 | 0.000 | -0.489 | 0.040 | 0.000 |
| Race3* | -0.219 | | 0.056 | 0.000 | -0.239 | 0.048 | 0.000 | -0.280 | 0.048 | 0.000 |
| Race4 | -0.020 | | 0.045 | 0.653 | -0.084 | 0.044 | 0.057 | -0.037 | 0.041 | 0.369 |
| Educ2 | 0.195 | | 0.134 | 0.145 | 0.117 | 0.113 | 0.301 | 0.025 | 0.122 | 0.837 |
| Educ3 | 0.258 | | 0.133 | 0.052 | 0.173 | 0.113 | 0.126 | 0.100 | 0.121 | 0.407 |
| Educ4 | 0.273 | | 0.140 | 0.050 | 0.234 | 0.115 | 0.043 | 0.168 | 0.123 | 0.172 |
| MS2* | 0.191 | | 0.051 | 0.000 | 0.252 | 0.051 | 0.000 | 0.284 | 0.052 | 0.000 |
| MS3* | 0.211 | | 0.031 | 0.000 | 0.252 | 0.030 | 0.000 | 0.228 | 0.032 | 0.000 |
| MS4* | 0.198 | | 0.067 | 0.003 | 0.325 | 0.063 | 0.000 | 0.186 | 0.065 | 0.004 |
| MS5* | 0.168 | | 0.030 | 0.000 | 0.178 | 0.028 | 0.000 | 0.227 | 0.029 | 0.000 |
| Numper* | -0.024 | | 0.009 | 0.006 | -0.024 | 0.010 | 0.010 | -0.001 | 0.010 | 0.948 |
| Numkid* | -0.106 | | 0.019 | 0.000 | -0.115 | 0.014 | 0.000 | -0.093 | 0.017 | 0.000 |
| Income2 | 0.000 | | 0.042 | 0.997 | 0.070 | 0.038 | 0.068 | 0.041 | 0.042 | 0.326 |
| Income3 | 0.007 | | 0.039 | 0.860 | 0.055 | 0.038 | 0.141 | 0.084 | 0.039 | 0.031 |
| Income4 | -0.002 | | 0.041 | 0.970 | 0.117 | 0.036 | 0.001 | 0.031 | 0.039 | 0.425 |
| Income5 | -0.008 | | 0.043 | 0.849 | 0.076 | 0.040 | 0.056 | 0.060 | 0.052 | 0.248 |
| Income6 | 0.049 | | 0.045 | 0.278 | 0.095 | 0.041 | 0.020 | 0.067 | 0.043 | 0.122 |
| Income7 | 0.016 | | 0.053 | 0.759 | 0.062 | 0.053 | 0.243 | 0.178 | 0.054 | 0.001 |
| Income8 | 0.011 | | 0.057 | 0.851 | 0.095 | 0.053 | 0.074 | 0.103 | 0.056 | 0.067 |
| Wrkloss* | 0.336 | | 0.023 | 0.000 | 0.337 | 0.021 | 0.000 | 0.298 | 0.023 | 0.000 |
| Anywork* | -0.134 | | 0.024 | 0.000 | -0.172 | 0.021 | 0.000 | -0.180 | 0.023 | 0.000 |
| Kindwork2 | 0.005 | | 0.046 | 0.918 | 0.026 | 0.036 | 0.463 | -0.077 | 0.038 | 0.041 |
| Kindwork3 | -0.041 | | 0.052 | 0.426 | 0.043 | 0.048 | 0.378 | -0.014 | 0.047 | 0.771 |
| Kindwork4 | 0.072 | | 0.050 | 0.154 | 0.081 | 0.040 | 0.043 | 0.024 | 0.042 | 0.571 |
| Kindwork5 | 0.007 | | 0.111 | 0.950 | 0.011 | 0.082 | 0.897 | 0.074 | 0.079 | 0.351 |
| Emppay | -0.083 | | 0.038 | 0.030 | 0.013 | 0.057 | 0.813 | -0.037 | 0.066 | 0.574 |
| Foodcon.change | -0.009 | | 0.047 | 0.851 | -0.061 | 0.045 | 0.176 | 0.014 | 0.051 | 0.783 |
| Freefood | 0.017 | | 0.037 | 0.641 | -0.038 | 0.038 | 0.321 | -0.036 | 0.037 | 0.329 |
| Tspndfood | 0.000 | | 0.000 | 0.004 | 0.000 | 0.000 | 0.004 | 0.000 | 0.000 | 0.809 |
| Tspndprpd | 0.000 | | 0.000 | 0.226 | 0.000 | 0.000 | 0.762 | 0.000 | 0.000 | 0.143 |
| Foodconf2* | -0.650 | | 0.039 | 0.000 | -0.598 | 0.038 | 0.000 | -0.606 | 0.038 | 0.000 |
| Foodconf3* | -0.900 | | 0.040 | 0.000 | -0.803 | 0.040 | 0.000 | -0.838 | 0.041 | 0.000 |
| Foodconf4* | -1.382 | | 0.043 | 0.000 | -1.316 | 0.044 | 0.000 | -1.370 | 0.043 | 0.000 |
| Hlthstatus2* | 0.386 | | 0.036 | 0.000 | 0.376 | 0.038 | 0.000 | 0.318 | 0.035 | 0.000 |
| Hlthstatus3* | 0.838 | | 0.036 | 0.000 | 0.821 | 0.037 | 0.000 | 0.789 | 0.036 | 0.000 |
| Hlthstatus4* | 1.483 | | 0.040 | 0.000 | 1.426 | 0.040 | 0.000 | 1.311 | 0.039 | 0.000 |
| Hlthstatus5* | 2.093 | | 0.065 | 0.000 | 2.061 | 0.062 | 0.000 | 1.955 | 0.060 | 0.000 |
| Healins* | -0.042 | | 0.039 | 0.285 | -0.024 | 0.037 | 0.522 | -0.062 | 0.039 | 0.115 |
| Med.delay.notget* | 0.725 | | 0.020 | 0.000 | 0.690 | 0.022 | 0.000 | 0.688 | 0.020 | 0.000 |
| Mort.prob* | 0.244 | | 0.028 | 0.000 | 0.256 | 0.034 | 0.000 | 0.226 | 0.027 | 0.000 |
| Schoolenroll* | 0.033 | | 0.057 | 0.562 | 0.085 | 0.034 | 0.011 | 0.121 | 0.036 | 0.001 |
| Ttch_Hrs | 0.002 | | 0.003 | 0.480 | 0.000 | 0.003 | 0.973 | 0.003 | 0.002 | 0.164 |
| * indicates the predictors are considered to be statistically significant in the sense that their p-values are smaller than 0.05 for more than 6 weeks. | | | | | | | | | | |

**Table S5.** Reduced Model: Point Estimates, Standard Errors and P-values of the Covariate Effects Individually Derived from the Data in Weeks 1-3

| Week  Covariate |  | 1 |  |  | 2 |  |  | 3 |  |
| --- | --- | --- | --- | --- | --- | --- | --- | --- | --- |
|  | Estimate | s.e. | p-value | Estimate | s.e. | p-value | Estimate | s.e. | p-value |
| (Intercept) | -0.124 | 0.083 | 0.134 | -0.103 | 0.118 | 0.381 | -0.146 | 0.070 | 0.038 |
| Age | -0.028 | 0.001 | 0.000 | -0.029 | 0.001 | 0.000 | -0.029 | 0.001 | 0.000 |
| Male | -0.296 | 0.024 | 0.000 | -0.212 | 0.032 | 0.000 | -0.279 | 0.019 | 0.000 |
| MS2 | 0.181 | 0.060 | 0.003 | 0.162 | 0.078 | 0.038 | 0.180 | 0.051 | 0.001 |
| MS3 | 0.267 | 0.031 | 0.000 | 0.306 | 0.042 | 0.000 | 0.261 | 0.026 | 0.000 |
| MS4 | 0.262 | 0.073 | 0.000 | 0.262 | 0.109 | 0.016 | 0.202 | 0.059 | 0.001 |
| MS5 | 0.107 | 0.030 | 0.000 | 0.089 | 0.042 | 0.034 | 0.117 | 0.025 | 0.000 |
| Numkid | -0.116 | 0.012 | 0.000 | -0.130 | 0.016 | 0.000 | -0.101 | 0.011 | 0.000 |
| Wrkloss | 0.331 | 0.026 | 0.000 | 0.366 | 0.033 | 0.000 | 0.378 | 0.019 | 0.000 |
| Anywork | -0.097 | 0.024 | 0.000 | -0.128 | 0.032 | 0.000 | -0.148 | 0.018 | 0.000 |
| Foodconf2 | -0.633 | 0.042 | 0.000 | -0.522 | 0.058 | 0.000 | -0.609 | 0.033 | 0.000 |
| Foodconf3 | -0.834 | 0.046 | 0.000 | -0.721 | 0.064 | 0.000 | -0.756 | 0.037 | 0.000 |
| Foodconf4 | -1.247 | 0.048 | 0.000 | -1.311 | 0.069 | 0.000 | -1.285 | 0.037 | 0.000 |
| Hlthstatus2 | 0.273 | 0.036 | 0.000 | 0.329 | 0.049 | 0.000 | 0.325 | 0.028 | 0.000 |
| Hlthstatus3 | 0.671 | 0.035 | 0.000 | 0.770 | 0.049 | 0.000 | 0.751 | 0.028 | 0.000 |
| Hlthstatus4 | 1.285 | 0.042 | 0.000 | 1.307 | 0.057 | 0.000 | 1.306 | 0.033 | 0.000 |
| Hlthstatus5 | 1.808 | 0.064 | 0.000 | 1.969 | 0.090 | 0.000 | 1.917 | 0.050 | 0.000 |
| Healins | -0.096 | 0.041 | 0.020 | -0.157 | 0.057 | 0.006 | -0.113 | 0.038 | 0.003 |
| Med.delay.notget | 0.636 | 0.023 | 0.000 | 0.695 | 0.031 | 0.000 | 0.697 | 0.018 | 0.000 |
| Mort.prob | 0.270 | 0.033 | 0.000 | 0.163 | 0.042 | 0.000 | 0.205 | 0.036 | 0.000 |

**Table S5 (continued).** Reduced Model: Point Estimates, Standard Errors and P-values of the Covariate Effects Individually Derived from the Data in Weeks 4-6

| Week  Covariate |  | 4 |  |  | 5 |  |  | 6 |  |
| --- | --- | --- | --- | --- | --- | --- | --- | --- | --- |
|  | Estimate | s.e. | p-value | Estimate | s.e. | p-value | Estimate | s.e. | p-value |
| (Intercept) | -0.027 | 0.082 | 0.742 | -0.084 | 0.077 | 0.278 | -0.023 | 0.094 | 0.804 |
| Age | -0.030 | 0.001 | 0.000 | -0.031 | 0.001 | 0.000 | -0.032 | 0.001 | 0.000 |
| Male | -0.253 | 0.021 | 0.000 | -0.257 | 0.023 | 0.000 | -0.233 | 0.023 | 0.000 |
| MS2 | 0.281 | 0.050 | 0.000 | 0.202 | 0.052 | 0.000 | 0.185 | 0.056 | 0.001 |
| MS3 | 0.270 | 0.028 | 0.000 | 0.269 | 0.027 | 0.000 | 0.296 | 0.032 | 0.000 |
| MS4 | 0.225 | 0.066 | 0.001 | 0.187 | 0.065 | 0.004 | 0.161 | 0.069 | 0.021 |
| MS5 | 0.171 | 0.028 | 0.000 | 0.233 | 0.026 | 0.000 | 0.224 | 0.031 | 0.000 |
| Numkid | -0.105 | 0.011 | 0.000 | -0.122 | 0.011 | 0.000 | -0.132 | 0.012 | 0.000 |
| Wrkloss | 0.380 | 0.021 | 0.000 | 0.344 | 0.021 | 0.000 | 0.349 | 0.023 | 0.000 |
| Anywork | -0.119 | 0.022 | 0.000 | -0.112 | 0.023 | 0.000 | -0.156 | 0.025 | 0.000 |
| Foodconf2 | -0.593 | 0.045 | 0.000 | -0.638 | 0.037 | 0.000 | -0.630 | 0.043 | 0.000 |
| Foodconf3 | -0.840 | 0.043 | 0.000 | -0.876 | 0.041 | 0.000 | -0.813 | 0.048 | 0.000 |
| Foodconf4 | -1.362 | 0.046 | 0.000 | -1.343 | 0.042 | 0.000 | -1.271 | 0.047 | 0.000 |
| Hlthstatus2 | 0.301 | 0.036 | 0.000 | 0.411 | 0.033 | 0.000 | 0.365 | 0.036 | 0.000 |
| Hlthstatus3 | 0.756 | 0.032 | 0.000 | 0.832 | 0.033 | 0.000 | 0.837 | 0.036 | 0.000 |
| Hlthstatus4 | 1.362 | 0.037 | 0.000 | 1.431 | 0.037 | 0.000 | 1.411 | 0.042 | 0.000 |
| Hlthstatus5 | 2.061 | 0.056 | 0.000 | 2.093 | 0.061 | 0.000 | 2.055 | 0.061 | 0.000 |
| Healins | -0.146 | 0.042 | 0.001 | -0.064 | 0.034 | 0.061 | -0.061 | 0.043 | 0.154 |
| Med.delay.notget | 0.692 | 0.021 | 0.000 | 0.687 | 0.020 | 0.000 | 0.744 | 0.022 | 0.000 |
| Mort.prob | 0.164 | 0.028 | 0.000 | 0.208 | 0.031 | 0.000 | 0.195 | 0.033 | 0.000 |

**Table S5 (continued).** Reduced Model: Point Estimates, Standard Errors and P-values of the Covariate Effects Individually Derived from the Data in Weeks 7-9

| Week  Covariate |  | 7 |  |  | 8 |  |  | 9 |  |
| --- | --- | --- | --- | --- | --- | --- | --- | --- | --- |
|  | Estimate | s.e. | p-value | Estimate | s.e. | p-value | Estimate | s.e. | p-value |
| (Intercept) | -0.060 | 0.088 | 0.495 | 0.018 | 0.072 | 0.801 | -0.073 | 0.074 | 0.324 |
| Age | -0.029 | 0.001 | 0.000 | -0.030 | 0.001 | 0.000 | -0.029 | 0.001 | 0.000 |
| Male | -0.190 | 0.024 | 0.000 | -0.211 | 0.020 | 0.000 | -0.186 | 0.020 | 0.000 |
| MS2 | 0.167 | 0.057 | 0.003 | 0.219 | 0.049 | 0.000 | 0.209 | 0.049 | 0.000 |
| MS3 | 0.285 | 0.031 | 0.000 | 0.238 | 0.026 | 0.000 | 0.200 | 0.028 | 0.000 |
| MS4 | 0.323 | 0.074 | 0.000 | 0.300 | 0.061 | 0.000 | 0.197 | 0.061 | 0.001 |
| MS5 | 0.204 | 0.032 | 0.000 | 0.136 | 0.025 | 0.000 | 0.096 | 0.026 | 0.000 |
| Numkid | -0.125 | 0.012 | 0.000 | -0.134 | 0.011 | 0.000 | -0.130 | 0.010 | 0.000 |
| Wrkloss | 0.378 | 0.025 | 0.000 | 0.330 | 0.022 | 0.000 | 0.387 | 0.021 | 0.000 |
| Anywork | -0.158 | 0.025 | 0.000 | -0.103 | 0.020 | 0.000 | -0.152 | 0.021 | 0.000 |
| Foodconf2 | -0.560 | 0.048 | 0.000 | -0.644 | 0.035 | 0.000 | -0.629 | 0.034 | 0.000 |
| Foodconf3 | -0.816 | 0.050 | 0.000 | -0.867 | 0.038 | 0.000 | -0.845 | 0.038 | 0.000 |
| Foodconf4 | -1.285 | 0.052 | 0.000 | -1.364 | 0.038 | 0.000 | -1.345 | 0.041 | 0.000 |
| Hlthstatus2 | 0.261 | 0.037 | 0.000 | 0.335 | 0.034 | 0.000 | 0.345 | 0.032 | 0.000 |
| Hlthstatus3 | 0.729 | 0.037 | 0.000 | 0.787 | 0.032 | 0.000 | 0.780 | 0.032 | 0.000 |
| Hlthstatus4 | 1.326 | 0.042 | 0.000 | 1.399 | 0.035 | 0.000 | 1.406 | 0.037 | 0.000 |
| Hlthstatus5 | 1.999 | 0.064 | 0.000 | 2.101 | 0.055 | 0.000 | 2.112 | 0.057 | 0.000 |
| Healins | -0.122 | 0.043 | 0.005 | -0.106 | 0.035 | 0.003 | -0.048 | 0.035 | 0.169 |
| Med.delay.notget | 0.669 | 0.025 | 0.000 | 0.677 | 0.019 | 0.000 | 0.729 | 0.020 | 0.000 |
| Mort.prob | 0.202 | 0.038 | 0.000 | 0.174 | 0.028 | 0.000 | 0.192 | 0.030 | 0.000 |

**Table S5 (continued).** Reduced Model: Point Estimates, Standard Errors and P-values of the Covariate Effects Individually Derived from the Data in Weeks 10-12

| Week  Covariate |  | 10 |  |  | 11 |  |  | 12 |  |
| --- | --- | --- | --- | --- | --- | --- | --- | --- | --- |
|  | Estimate | s.e. | p-value | Estimate | s.e. | p-value | Estimate | s.e. | p-value |
| (Intercept) | -0.130 | 0.085 | 0.129 | -0.054 | 0.078 | 0.488 | 0.079 | 0.077 | 0.307 |
| Age | -0.029 | 0.001 | 0.000 | -0.030 | 0.001 | 0.000 | -0.029 | 0.001 | 0.000 |
| Male | -0.167 | 0.021 | 0.000 | -0.168 | 0.021 | 0.000 | -0.206 | 0.022 | 0.000 |
| MS2 | 0.178 | 0.050 | 0.000 | 0.212 | 0.051 | 0.000 | 0.237 | 0.051 | 0.000 |
| MS3 | 0.203 | 0.029 | 0.000 | 0.236 | 0.028 | 0.000 | 0.205 | 0.029 | 0.000 |
| MS4 | 0.141 | 0.066 | 0.034 | 0.257 | 0.062 | 0.000 | 0.123 | 0.063 | 0.052 |
| MS5 | 0.126 | 0.029 | 0.000 | 0.110 | 0.026 | 0.000 | 0.156 | 0.027 | 0.000 |
| Numkid | -0.128 | 0.011 | 0.000 | -0.132 | 0.010 | 0.000 | -0.084 | 0.011 | 0.000 |
| Wrkloss | 0.343 | 0.022 | 0.000 | 0.342 | 0.021 | 0.000 | 0.308 | 0.021 | 0.000 |
| Anywork | -0.129 | 0.023 | 0.000 | -0.154 | 0.021 | 0.000 | -0.161 | 0.021 | 0.000 |
| Foodconf2 | -0.635 | 0.039 | 0.000 | -0.571 | 0.038 | 0.000 | -0.592 | 0.037 | 0.000 |
| Foodconf3 | -0.864 | 0.039 | 0.000 | -0.752 | 0.039 | 0.000 | -0.799 | 0.040 | 0.000 |
| Foodconf4 | -1.313 | 0.041 | 0.000 | -1.234 | 0.042 | 0.000 | -1.283 | 0.041 | 0.000 |
| Hlthstatus2 | 0.378 | 0.036 | 0.000 | 0.365 | 0.038 | 0.000 | 0.306 | 0.034 | 0.000 |
| Hlthstatus3 | 0.807 | 0.035 | 0.000 | 0.787 | 0.038 | 0.000 | 0.748 | 0.035 | 0.000 |
| Hlthstatus4 | 1.435 | 0.040 | 0.000 | 1.371 | 0.039 | 0.000 | 1.250 | 0.038 | 0.000 |
| Hlthstatus5 | 2.042 | 0.064 | 0.000 | 1.997 | 0.063 | 0.000 | 1.880 | 0.058 | 0.000 |
| Healins | -0.037 | 0.038 | 0.322 | -0.018 | 0.036 | 0.626 | -0.052 | 0.039 | 0.183 |
| Med.delay.notget | 0.752 | 0.020 | 0.000 | 0.717 | 0.021 | 0.000 | 0.723 | 0.020 | 0.000 |
| Mort.prob | 0.206 | 0.028 | 0.000 | 0.207 | 0.032 | 0.000 | 0.173 | 0.027 | 0.000 |

**Table S6.** Final Model: Point Estimates, Standard Errors and P-values of the Covariate Effects Individually Derived from the Data in Weeks 1-3

| Week  Covariate |  | 1 |  |  | 2 |  |  | 3 |  |
| --- | --- | --- | --- | --- | --- | --- | --- | --- | --- |
|  | Estimate | s.e. | p-value | Estimate | s.e. | p-value | Estimate | s.e. | p-value |
| (Intercept) | 0.075 | 0.094 | 0.427 | 0.006 | 0.130 | 0.962 | 0.072 | 0.078 | 0.353 |
| State.mild | -0.095 | 0.032 | 0.003 | -0.085 | 0.043 | 0.048 | -0.107 | 0.023 | 0.000 |
| State.moderate.daily | -0.019 | 0.046 | 0.678 | 0.057 | 0.064 | 0.375 | -0.088 | 0.035 | 0.011 |
| State.serious | 0.188 | 0.074 | 0.011 | 0.135 | 0.102 | 0.185 | 0.011 | 0.058 | 0.854 |
| Age | -0.029 | 0.001 | 0.000 | -0.030 | 0.001 | 0.000 | -0.029 | 0.001 | 0.000 |
| Male | -0.303 | 0.025 | 0.000 | -0.216 | 0.032 | 0.000 | -0.284 | 0.019 | 0.000 |
| Rhispanic | -0.148 | 0.047 | 0.002 | -0.140 | 0.058 | 0.015 | -0.131 | 0.032 | 0.000 |
| Race2 | -0.450 | 0.039 | 0.000 | -0.360 | 0.058 | 0.000 | -0.474 | 0.031 | 0.000 |
| Race3 | -0.214 | 0.063 | 0.001 | -0.279 | 0.080 | 0.001 | -0.225 | 0.045 | 0.000 |
| Race4 | -0.074 | 0.049 | 0.134 | -0.097 | 0.068 | 0.151 | -0.045 | 0.044 | 0.306 |
| MS2 | 0.185 | 0.062 | 0.003 | 0.185 | 0.080 | 0.021 | 0.189 | 0.054 | 0.000 |
| MS3 | 0.249 | 0.032 | 0.000 | 0.310 | 0.044 | 0.000 | 0.242 | 0.027 | 0.000 |
| MS4 | 0.277 | 0.073 | 0.000 | 0.288 | 0.109 | 0.008 | 0.222 | 0.059 | 0.000 |
| MS5 | 0.149 | 0.031 | 0.000 | 0.128 | 0.043 | 0.003 | 0.150 | 0.026 | 0.000 |
| Numper | -0.026 | 0.012 | 0.029 | 0.011 | 0.016 | 0.484 | -0.029 | 0.008 | 0.001 |
| Numkid | -0.098 | 0.021 | 0.000 | -0.143 | 0.024 | 0.000 | -0.085 | 0.015 | 0.000 |
| Wrkloss | 0.328 | 0.026 | 0.000 | 0.352 | 0.034 | 0.000 | 0.377 | 0.020 | 0.000 |
| Anywork | -0.100 | 0.025 | 0.000 | -0.133 | 0.033 | 0.000 | -0.157 | 0.019 | 0.000 |
| Foodconf2 | -0.651 | 0.042 | 0.000 | -0.529 | 0.059 | 0.000 | -0.627 | 0.032 | 0.000 |
| Foodconf3 | -0.866 | 0.047 | 0.000 | -0.746 | 0.065 | 0.000 | -0.791 | 0.036 | 0.000 |
| Foodconf4 | -1.296 | 0.049 | 0.000 | -1.344 | 0.070 | 0.000 | -1.337 | 0.037 | 0.000 |
| Hlthstatus2 | 0.276 | 0.036 | 0.000 | 0.328 | 0.050 | 0.000 | 0.327 | 0.028 | 0.000 |
| Hlthstatus3 | 0.682 | 0.035 | 0.000 | 0.779 | 0.050 | 0.000 | 0.762 | 0.029 | 0.000 |
| Hlthstatus4 | 1.307 | 0.042 | 0.000 | 1.319 | 0.057 | 0.000 | 1.324 | 0.033 | 0.000 |
| Hlthstatus5 | 1.834 | 0.064 | 0.000 | 1.983 | 0.091 | 0.000 | 1.933 | 0.050 | 0.000 |
| Healins | -0.092 | 0.041 | 0.026 | -0.152 | 0.058 | 0.009 | -0.108 | 0.038 | 0.004 |
| Med.delay.notget | 0.622 | 0.023 | 0.000 | 0.685 | 0.031 | 0.000 | 0.678 | 0.019 | 0.000 |
| Mort.prob | 0.305 | 0.033 | 0.000 | 0.192 | 0.043 | 0.000 | 0.241 | 0.037 | 0.000 |
| Schoolenroll | 0.104 | 0.045 | 0.020 | 0.071 | 0.058 | 0.217 | 0.126 | 0.030 | 0.000 |

**Table S6 (continued).** Final Model: Point Estimates, Standard Errors and P-values of the Covariate Effects Individually Derived from the Data in Weeks 4-6

| Week  Covariate |  | 4 |  |  | 5 |  |  | 6 |  |
| --- | --- | --- | --- | --- | --- | --- | --- | --- | --- |
|  | Estimate | s.e. | p-value | Estimate | s.e. | p-value | Estimate | s.e. | p-value |
| (Intercept) | 0.184 | 0.089 | 0.040 | 0.226 | 0.087 | 0.009 | 0.228 | 0.099 | 0.022 |
| State.mild | -0.099 | 0.028 | 0.000 | -0.183 | 0.027 | 0.000 | -0.178 | 0.030 | 0.000 |
| State.moderate.daily | -0.040 | 0.041 | 0.340 | -0.076 | 0.042 | 0.070 | -0.074 | 0.043 | 0.081 |
| State.serious | -0.038 | 0.068 | 0.574 | -0.161 | 0.067 | 0.016 | 0.020 | 0.074 | 0.790 |
| Age | -0.031 | 0.001 | 0.000 | -0.032 | 0.001 | 0.000 | -0.033 | 0.001 | 0.000 |
| Male | -0.261 | 0.021 | 0.000 | -0.259 | 0.024 | 0.000 | -0.238 | 0.023 | 0.000 |
| Rhispanic | -0.124 | 0.037 | 0.001 | -0.190 | 0.034 | 0.000 | -0.196 | 0.039 | 0.000 |
| Race2 | -0.503 | 0.039 | 0.000 | -0.420 | 0.037 | 0.000 | -0.443 | 0.041 | 0.000 |
| Race3 | -0.306 | 0.052 | 0.000 | -0.362 | 0.049 | 0.000 | -0.297 | 0.060 | 0.000 |
| Race4 | 0.065 | 0.046 | 0.160 | -0.079 | 0.050 | 0.116 | -0.004 | 0.051 | 0.945 |
| MS2 | 0.284 | 0.051 | 0.000 | 0.205 | 0.053 | 0.000 | 0.201 | 0.055 | 0.000 |
| MS3 | 0.245 | 0.029 | 0.000 | 0.242 | 0.028 | 0.000 | 0.273 | 0.032 | 0.000 |
| MS4 | 0.246 | 0.067 | 0.000 | 0.200 | 0.065 | 0.002 | 0.174 | 0.071 | 0.014 |
| MS5 | 0.197 | 0.028 | 0.000 | 0.260 | 0.027 | 0.000 | 0.256 | 0.032 | 0.000 |
| Numper | -0.035 | 0.009 | 0.000 | -0.036 | 0.009 | 0.000 | -0.028 | 0.010 | 0.005 |
| Numkid | -0.085 | 0.018 | 0.000 | -0.099 | 0.017 | 0.000 | -0.126 | 0.018 | 0.000 |
| Wrkloss | 0.383 | 0.021 | 0.000 | 0.345 | 0.021 | 0.000 | 0.342 | 0.023 | 0.000 |
| Anywork | -0.127 | 0.023 | 0.000 | -0.122 | 0.022 | 0.000 | -0.168 | 0.026 | 0.000 |
| Foodconf2 | -0.604 | 0.045 | 0.000 | -0.654 | 0.037 | 0.000 | -0.649 | 0.043 | 0.000 |
| Foodconf3 | -0.865 | 0.043 | 0.000 | -0.909 | 0.040 | 0.000 | -0.843 | 0.048 | 0.000 |
| Foodconf4 | -1.404 | 0.045 | 0.000 | -1.397 | 0.042 | 0.000 | -1.315 | 0.046 | 0.000 |
| Hlthstatus2 | 0.303 | 0.036 | 0.000 | 0.416 | 0.033 | 0.000 | 0.369 | 0.036 | 0.000 |
| Hlthstatus3 | 0.767 | 0.032 | 0.000 | 0.847 | 0.033 | 0.000 | 0.849 | 0.036 | 0.000 |
| Hlthstatus4 | 1.384 | 0.037 | 0.000 | 1.456 | 0.037 | 0.000 | 1.437 | 0.043 | 0.000 |
| Hlthstatus5 | 2.076 | 0.055 | 0.000 | 2.112 | 0.060 | 0.000 | 2.074 | 0.062 | 0.000 |
| Healins | -0.140 | 0.043 | 0.001 | -0.058 | 0.035 | 0.096 | -0.057 | 0.042 | 0.176 |
| Med.delay.notget | 0.670 | 0.021 | 0.000 | 0.666 | 0.020 | 0.000 | 0.726 | 0.022 | 0.000 |
| Mort.prob | 0.202 | 0.029 | 0.000 | 0.242 | 0.032 | 0.000 | 0.232 | 0.034 | 0.000 |
| Schoolenroll | 0.119 | 0.041 | 0.004 | 0.126 | 0.033 | 0.000 | 0.186 | 0.042 | 0.000 |

**Table S6 (continued).** Final Model: Point Estimates, Standard Errors and P-values of the Covariate Effects Individually Derived from the Data in Weeks 7-9

| Week  Covariate |  | 7 |  |  | 8 |  |  | 9 |  |
| --- | --- | --- | --- | --- | --- | --- | --- | --- | --- |
|  | Estimate | s.e. | p-value | Estimate | s.e. | p-value | Estimate | s.e. | p-value |
| (Intercept) | 0.179 | 0.095 | 0.058 | 0.268 | 0.078 | 0.001 | 0.177 | 0.078 | 0.024 |
| State.mild | -0.162 | 0.030 | 0.000 | -0.139 | 0.026 | 0.000 | -0.129 | 0.025 | 0.000 |
| State.moderate.daily | -0.075 | 0.043 | 0.082 | -0.011 | 0.036 | 0.770 | -0.022 | 0.038 | 0.561 |
| State.serious | -0.133 | 0.075 | 0.077 | -0.059 | 0.065 | 0.366 | -0.101 | 0.066 | 0.128 |
| Age | -0.030 | 0.001 | 0.000 | -0.030 | 0.001 | 0.000 | -0.030 | 0.001 | 0.000 |
| Male | -0.195 | 0.024 | 0.000 | -0.217 | 0.020 | 0.000 | -0.194 | 0.020 | 0.000 |
| Rhispanic | -0.246 | 0.043 | 0.000 | -0.174 | 0.035 | 0.000 | -0.171 | 0.037 | 0.000 |
| Race2 | -0.406 | 0.042 | 0.000 | -0.409 | 0.032 | 0.000 | -0.412 | 0.038 | 0.000 |
| Race3 | -0.276 | 0.055 | 0.000 | -0.275 | 0.046 | 0.000 | -0.232 | 0.051 | 0.000 |
| Race4 | -0.090 | 0.053 | 0.091 | -0.016 | 0.039 | 0.689 | -0.022 | 0.040 | 0.587 |
| MS2 | 0.181 | 0.058 | 0.002 | 0.214 | 0.049 | 0.000 | 0.196 | 0.051 | 0.000 |
| MS3 | 0.273 | 0.032 | 0.000 | 0.210 | 0.027 | 0.000 | 0.173 | 0.028 | 0.000 |
| MS4 | 0.348 | 0.075 | 0.000 | 0.313 | 0.060 | 0.000 | 0.215 | 0.062 | 0.001 |
| MS5 | 0.241 | 0.032 | 0.000 | 0.158 | 0.026 | 0.000 | 0.120 | 0.026 | 0.000 |
| Numper | -0.014 | 0.010 | 0.177 | -0.039 | 0.009 | 0.000 | -0.041 | 0.009 | 0.000 |
| Numkid | -0.125 | 0.021 | 0.000 | -0.111 | 0.014 | 0.000 | -0.094 | 0.015 | 0.000 |
| Wrkloss | 0.373 | 0.025 | 0.000 | 0.333 | 0.022 | 0.000 | 0.395 | 0.022 | 0.000 |
| Anywork | -0.167 | 0.026 | 0.000 | -0.114 | 0.020 | 0.000 | -0.157 | 0.020 | 0.000 |
| Foodconf2 | -0.570 | 0.049 | 0.000 | -0.659 | 0.035 | 0.000 | -0.642 | 0.035 | 0.000 |
| Foodconf3 | -0.840 | 0.051 | 0.000 | -0.893 | 0.038 | 0.000 | -0.868 | 0.039 | 0.000 |
| Foodconf4 | -1.329 | 0.052 | 0.000 | -1.408 | 0.038 | 0.000 | -1.386 | 0.041 | 0.000 |
| Hlthstatus2 | 0.265 | 0.037 | 0.000 | 0.336 | 0.033 | 0.000 | 0.345 | 0.032 | 0.000 |
| Hlthstatus3 | 0.742 | 0.037 | 0.000 | 0.796 | 0.032 | 0.000 | 0.791 | 0.033 | 0.000 |
| Hlthstatus4 | 1.350 | 0.042 | 0.000 | 1.418 | 0.035 | 0.000 | 1.428 | 0.037 | 0.000 |
| Hlthstatus5 | 2.022 | 0.064 | 0.000 | 2.117 | 0.054 | 0.000 | 2.129 | 0.057 | 0.000 |
| Healins | -0.125 | 0.044 | 0.004 | -0.101 | 0.035 | 0.004 | -0.046 | 0.035 | 0.186 |
| Med.delay.notget | 0.650 | 0.025 | 0.000 | 0.658 | 0.019 | 0.000 | 0.710 | 0.021 | 0.000 |
| Mort.prob | 0.240 | 0.039 | 0.000 | 0.210 | 0.028 | 0.000 | 0.227 | 0.031 | 0.000 |
| Schoolenroll | 0.121 | 0.060 | 0.042 | 0.136 | 0.023 | 0.000 | 0.079 | 0.036 | 0.029 |

**Table S6 (continued).** Final Model: Point Estimates, Standard Errors and P-values of the Covariate Effects Individually Derived from the Data in Weeks 10-12

| Week  Covariate |  | 10 |  |  | 11 |  |  | 12 |  |
| --- | --- | --- | --- | --- | --- | --- | --- | --- | --- |
|  | Estimate | s.e. | p-value | Estimate | s.e. | p-value | Estimate | s.e. | p-value |
| (Intercept) | 0.118 | 0.088 | 0.181 | 0.207 | 0.084 | 0.014 | 0.253 | 0.086 | 0.004 |
| State.mild | -0.143 | 0.027 | 0.000 | -0.206 | 0.026 | 0.000 | -0.148 | 0.027 | 0.000 |
| State.moderate.daily | -0.054 | 0.039 | 0.159 | -0.168 | 0.040 | 0.000 | -0.068 | 0.039 | 0.085 |
| State.serious | 0.023 | 0.069 | 0.742 | -0.152 | 0.068 | 0.025 | -0.197 | 0.069 | 0.004 |
| Age | -0.029 | 0.001 | 0.000 | -0.031 | 0.001 | 0.000 | -0.029 | 0.001 | 0.000 |
| Male | -0.178 | 0.022 | 0.000 | -0.176 | 0.021 | 0.000 | -0.213 | 0.022 | 0.000 |
| Rhispanic | -0.250 | 0.037 | 0.000 | -0.144 | 0.035 | 0.000 | -0.154 | 0.035 | 0.000 |
| Race2 | -0.481 | 0.038 | 0.000 | -0.508 | 0.038 | 0.000 | -0.492 | 0.038 | 0.000 |
| Race3 | -0.199 | 0.054 | 0.000 | -0.216 | 0.048 | 0.000 | -0.263 | 0.048 | 0.000 |
| Race4 | -0.024 | 0.042 | 0.577 | -0.090 | 0.042 | 0.034 | -0.039 | 0.041 | 0.339 |
| MS2 | 0.170 | 0.050 | 0.001 | 0.217 | 0.050 | 0.000 | 0.249 | 0.051 | 0.000 |
| MS3 | 0.193 | 0.029 | 0.000 | 0.226 | 0.030 | 0.000 | 0.198 | 0.031 | 0.000 |
| MS4 | 0.180 | 0.067 | 0.007 | 0.295 | 0.063 | 0.000 | 0.150 | 0.065 | 0.021 |
| MS5 | 0.157 | 0.028 | 0.000 | 0.152 | 0.027 | 0.000 | 0.204 | 0.027 | 0.000 |
| Numper | -0.023 | 0.009 | 0.009 | -0.022 | 0.009 | 0.015 | -0.005 | 0.009 | 0.626 |
| Numkid | -0.099 | 0.018 | 0.000 | -0.112 | 0.013 | 0.000 | -0.090 | 0.017 | 0.000 |
| Wrkloss | 0.349 | 0.023 | 0.000 | 0.343 | 0.021 | 0.000 | 0.302 | 0.022 | 0.000 |
| Anywork | -0.130 | 0.024 | 0.000 | -0.156 | 0.021 | 0.000 | -0.167 | 0.022 | 0.000 |
| Foodconf2 | -0.648 | 0.039 | 0.000 | -0.585 | 0.038 | 0.000 | -0.601 | 0.037 | 0.000 |
| Foodconf3 | -0.891 | 0.040 | 0.000 | -0.779 | 0.039 | 0.000 | -0.822 | 0.040 | 0.000 |
| Foodconf4 | -1.362 | 0.041 | 0.000 | -1.277 | 0.042 | 0.000 | -1.322 | 0.041 | 0.000 |
| Hlthstatus2 | 0.377 | 0.036 | 0.000 | 0.368 | 0.038 | 0.000 | 0.305 | 0.034 | 0.000 |
| Hlthstatus3 | 0.818 | 0.036 | 0.000 | 0.802 | 0.037 | 0.000 | 0.759 | 0.035 | 0.000 |
| Hlthstatus4 | 1.457 | 0.040 | 0.000 | 1.394 | 0.039 | 0.000 | 1.267 | 0.038 | 0.000 |
| Hlthstatus5 | 2.062 | 0.065 | 0.000 | 2.015 | 0.063 | 0.000 | 1.899 | 0.058 | 0.000 |
| Healins | -0.045 | 0.038 | 0.230 | -0.015 | 0.036 | 0.689 | -0.056 | 0.039 | 0.152 |
| Med.delay.notget | 0.737 | 0.020 | 0.000 | 0.702 | 0.021 | 0.000 | 0.705 | 0.020 | 0.000 |
| Mort.prob | 0.241 | 0.028 | 0.000 | 0.244 | 0.034 | 0.000 | 0.211 | 0.027 | 0.000 |
| Schoolenroll | 0.035 | 0.057 | 0.546 | 0.089 | 0.033 | 0.008 | 0.119 | 0.036 | 0.001 |
